# Non‐Equimolar High‐Entropy Spinel Oxides as Anode Materials for a Direct Ammonia‐Fed Solid Oxide Fuel Cell

**DOI:** 10.1002/smsc.70427

**Published:** 2026-10-21

**Authors:** Omer Elmutasim, Maxwell P. Pinczes, Dattatray S. Dhawale, Sarbjit Giddey, Sankar Bhattacharya

**Affiliations:** ^1^ CSIRO Energy Clayton South Victoria Australia; ^2^ Department of Chemical and Biological Engineering Monash University Clayton Victoria Australia

**Keywords:** (M_0.3_Ni_0.21_Cu_0.21_Co_0.14_Mn_0.14_)Fe_2_O_4_ (M = Fe, Mg, Zn), DA–SOFC, density functional theory, micro‐computed tomography, non–equimolar high‐entropy oxides, reaction mechanism, spinel

## Abstract

High‐entropy oxides (HEOs) have emerged as promising electrocatalysts owing to their unique cocktail effect, which enables synergistic multielement interactions, leading to enhanced catalytic activity. In this work, three novel non‐equimolar high‐entropy spinel ferrites‐based materials, (M_0.3_Ni_0.21_Cu_0.21_Co_0.14_Mn_0.14_)Fe_2_O_4_ (M = Fe, Mg, or Zn)‐Gd_0.1_Ce_0.9_O_1.95_ (GDC), have been adopted as anodes for direct–ammonia solid oxide fuel cells (DA–SOFCs). Synchrotron‐based X‐ray absorption spectroscopy (XAS) and micro‐computed tomography (Micro‐CT) were employed, alongside X‐ray diffraction (XRD), inductively coupled plasma‐optical emission spectroscopy (ICP‐OES), scanning electron microscopy (SEM), scanning transmission electron microscopy with energy‐dispersive X‐ray spectroscopy (STEM‐EDX), X‐ray photoelectron spectroscopy (XPS), and Raman spectroscopy, for comprehensive characterization. The XRD analysis showed that the HEO(Zn) and HEO(Mg) samples exhibited a single‐phase high‐entropy spinel ferrite, whereas the d‐HEO(Fe) displayed a dual‐phase structure comprising a predominant spinel phase (90.9 wt%) and a minor hematite (Fe_2_O_3_) phase. The HEO samples were treated in a reducing atmosphere to form a composite with a monometallic Fe phase (>82.4 wt%) and FeNiCu alloy. Extended X‐ray absorption fine structure spectroscopy (EXAFS) analysis revealed that the Fe^3+^, Cu^2+^, Ni^2+^, and Co^2+^ cations are distributed between the octahedral and tetrahedral coordination environments within d‐HEO(Fe) sample, while the Mn^3+^ ions are located at the octahedral sites. The lanthanum strontium gallium magnesium oxide (LSGM) electrolyte‐supported cell with a d‐HEO(Fe) anode achieved the highest peak power density (PPD) of nearly 382.0 mW/cm^2^ at 850 °C, whereas the SOFCs with HEO(Zn) and HEO(Mg) anodes delivered lower PPD values of 218.3 and 195.7 mW/cm^2^, respectively. Notably, the cell with the d‐HEO(Fe) anode demonstrated excellent durability for over 75 h at 800 °C, with only 3.16% voltage degradation. Density functional theory (DFT) computations were carried out to scrutinize the NH_3_ decomposition mechanism on the Fe(110) surface, which represents the reduced HEO anode, and were benchmarked against those on the Ni(111) surface. Interestingly, the formation of the N_2_* species via recombination of N^*^ atoms is identified as the rate‐controlling step for NH_3_ cracking on Fe(110) and Ni(111), exhibiting the highest activation barriers of 2.43 and 1.63 eV, respectively, among all elementary steps considered.

## Graphical abstract

Current‐voltage‐power density performance curves of the SOFCs incorporating the d‐HEO(Fe), HEO(Zn) and HEO(Mg)‐based anodes at temperature of 850 °C.

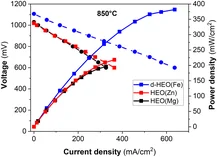

## 1 Introduction

Solid oxide fuel cell (SOFC) is a maturing technology that offers reliable and sustainable power generation with a plethora of applications ranging from kW‐ to GW‐scale power plants and auxiliary units [1, 2]. SOFCs leverage intermediate temperatures (600–800 °C) [3, 4, 5, 6] during operation for exceptional electrical efficiencies approaching upwards of 85% when combining excess heat utilisation systems [1, 7]. Additionally, this operational temperature allows for the direct use of various fuels such as ammonia, hydrocarbons, and syngas over the conventionally used hydrogen without externally processing the fuel first [8, 9, 10]. This advantage enables SOFCs to avoid the significant risks associated with the storage and transportation of hydrogen [11] and leverage the benefits that come with direct‐ammonia SOFCs (DA–SOFCs). DA–SOFCs have garnered significant attention through research within the past decade due to ammonia's exceptional volumetric energy density and volumetric hydrogen density [12]. Notably, ammonia can be stored and transported at near ambient temperatures [13] and can leverage beneficial thermal management through the exothermic electro‐oxidation and endothermic ammonia decomposition [14]. Directly using ammonia as the fuel does come with a significant challenge to anode stability when used with traditional Ni‐cermet anodes. When ammonia decomposes over Ni active sites, nitrogen reacts with nickel to form nickel nitrides (Ni_3_N). These compounds make the anode structure unstable, reduce its conductivity, and reduce the active catalyst sites [15].

Notably, conventional anodes struggle to capture the utility of DA–SOFCs due to sluggish catalytic activity towards N–H bond cleavage as well as anode degradation via the previously mentioned nickel nitrides. To combat these drawbacks, a new anode material would have to have superior catalytic action and stability towards nitridation without compromising on exceptional mixed electronic/ionic conductivity (MEIC) or the cost/simplicity of fabrication. A material that has increasingly shown these traits over the past decade is high‐entropy materials, specifically, high‐entropy oxides (HEO), in which five or more metals are held together in a crystal structure. Since HEO's conception in the research space in 2015 [16], it has seen significant use within catalytic literature [17], lithium battery anodes [18], and SOFC electrodes [19, 20, 21, 22, 23]. This widespread use is due to the “cocktail effect” in which different precursors or ratios can fine‐tune the characteristics of the material. Importantly, this research shows the ability to create materials that display excellent electrocatalytic performance for ammonia decomposition with remarkable stability [17], and survive the harsh conditions presented by SOFCs whilst retaining optimal conductivities [24]. Since this field of research is relatively new, the predominant focus has been on equimolar HEOs, in which the precursors have an equal ratio in synthesis [20, 21]. However, newer insights have shown that non‐equimolar HEOs, not an equal ratio of metals, display improved characteristics for electrode materials [24, 25, 26]. Additionally, there is a precedent for using both medium‐entropy materials and complex oxides through various perovskite anodes. Some notable examples include a medium‐entropy SrV_1/3_Fe_1/3_Mo_1/3_O_3_ (SVFMO) anode layer demonstrating 720 mW/cm^2^ at 850 °C with hydrogen fuel in which the SVFMO is attributed higher catalytic abilities due to the larger number of oxygen vacancies within the perovskite lattice as well as the exceptional electrical conductivity of the resulting reduced metal phase [27]. Another example that shows high catalytic activity is the Sr_1.9_Fe_1.5−*x*_Ni_*x*_Mo_0.5_O_6−δ_ (SFNM) anode that demonstrates 516 mW/cm^2^ at 800 °C with direct ammonia fueling. This high electrochemical performance is attributed to the exsolution of FeNi_3_ nanoparticles from the perovskite lattice to the surface of the anode [28]. Both of these complex oxide materials have sought to utilise the various decomposition mechanisms under reducing conditions to produce a more stable and active anode. Specifically, the exsolution of nanoparticles from the bulk lattice of the anode has produced significant advantages in performance and stability under operation [29, 30, 31]. The immense potential of combining materials that significantly enhance the electrocatalytic action, electrical conductivity, and stability of materials such as Ni, Cu, and Fe [32, 33, 34], combined with the precedent for medium‐entropy oxides as anode materials seen with the SVFMO and SFNM anodes, demonstrates that there is a significant gap in the literature surrounding HEO materials as SOFC anodes, as these same benefits and mechanisms could be extracted. To the best of the authors’ knowledge, there are no reports in the open literature addressing the use of non‐equimolar HEOs as anode materials for SOFCs. In this work, non‐equimolar HEO–GDC composites are employed as anode materials. Specifically, (M_0.3_Ni_0.21_Cu_0.21_Co_0.14_Mn_0.14_)Fe_2_O_4_, M = (Fe, Mg, and Zn), was prepared through the sol–gel. The molar ratio of M:Ni:Cu:Co:Mn = 0.30:0.21:0.21:0.14:0.14:2.00 was deliberately selected to increase the concentration of Fe, Ni, and Cu transition metals, which are known to exhibit catalytic activity toward ammonia cracking, while simultaneously promoting high electrical conductivity of the anode material. These anodes were utilised over an lanthanum strontium gallium magnesium (LSGM) electrolyte‐supported cell between 700 and 850 °C, and their performance and stability with ammonia as the fuel were evaluated. The HEO samples were thoroughly characterized via Synchrotron‐based X‐ray absorption spectroscopy (XAS) and micro‐computed tomography (Micro‐CT), XRD, inductively coupled plasma‐optical emission spectroscopy (ICP‐OES), scanning electron microscopy (SEM), scanning transmission electron microscopy with energy‐dispersive X‐ray spectroscopy (STEM‐EDX), X‐ray photoelectron spectroscopy (XPS), and Raman spectroscopy. Furthermore, density functional theory (DFT) computations were carried out to scrutinize the NH_3_ decomposition mechanism on the Fe(110) surface, representing the reduced HEO anode, and benchmarked against the Ni(111) surface. This work provides a key foothold into the window of HEO anode materials and a foundation for further investigation on optimising non‐equimolar HEOs.

## 2 Methodology

### 2.1 Materials

The LSGM electrolyte slurry was prepared using La_0.8_Sr_0.2_Ga_0.8_Mg_0.2_O_2.8_ powder (LSGM8282, Kceracell, Republic of Korea), while Gd_0.1_Ce_0.9_O_1.95_ (GDC10) powder and La_0.4_Ce_0.6_O_1.8_ (LDC) paste were procured from Kceracell (Republic of Korea). An aqueous emulsion system (Polymer Innovations Inc., USA), consists of acrylic binder (WB4101), dispersant (DS001), nonsilicone defoamer (DF002), and polymetric thickening agents (TK004 & TK005), is used. The precursors used for the synthesis of the HEO powders were: zinc nitrate hexahydrate (Zn(NO_3_)_2_·6H_2_O), manganese (II) nitrate tetrahydrate (Mn(NO_3_)_2_·4H_2_O), cobalt (II) nitrate hexahydrate (Co(NO_3_)_2_·6H_2_O), magnesium nitrate hexahydrate (Mg(NO_3_)_2_·6H_2_O), iron (II) sulfate heptahydrate (FeSO_4_·7H_2_O), nickel (II) nitrate hexahydrate (Ni (NO_3_)_2_·6 H_2_O), copper (II) nitrate trihydrate (Cu (NO_3_)_2_·3 H_2_O), and iron (III) nitrate nonahydrate (Fe (NO_3_)_3_·9 H_2_O), all produced by Sigma–Aldrich as well as glycine (H_2_NCH_2_COOH) produced by Merck.

### 2.2 Synthesis

The sol–gel technique was followed for the synthesis of the HEO samples. Precursors of Co(NO_3_)_2_·6H_2_O, Mn(NO_3_)_2_·4H_2_O, Cu(NO_3_)_2_·3H_2_O, Ni(NO_3_)_2_·6H_2_O and Fe(NO_3_)_3_· 9H_2_O were used in conjunction with either Zn(NO_3_)_2_·6H_2_O, Mg(NO_3_)_2_·6H_2_O, or FeSO_4_·7H_2_O to create (M_0.3_Ni_0.21_Cu_0.21_Co_0.14_Mn_0.14_)Fe_2_O_4,_ with M = Fe, Mg, or Zn. Stoichiometric amounts of the precursors were thoroughly mixed for 30 min under magnetic stirring to ensure a molar ratio of 0.30:0.21:0.21:0.14:0.14:2.00, corresponding to M:Ni:Cu:Co:Mn:Fe. Glycine was added at a molar ratio of 1:2 for the ratio of cations:glycine. This solution is then stirred at 80 °C to produce a gel. Once gelification occurred, the resultant gel was initially dried at 120 °C, followed by combustion at 300 °C for 12 h using a furnace. Various powders were then obtained and their phases were purified through annealing at 900 °C for both Mg_0.3_Ni_0.21_Cu_0.21_Co_0.14_Mn_0.14_) Fe_2_O_4_ (hereafter denoted as HEO(Mg)) and Zn_0.3_Ni_0.21_Cu_0.21_Co_0.14_Mn_0.14_) Fe_2_O_4_ (hereafter referred to as HEO(Zn)), while (Fe_0.3_Ni_0.21_Cu_0.21_Co_0.14_Mn_0.14_) Fe_2_O_4_ (hereafter denoted as d‐HEO(Fe)) was sintered at 1300 °C.

### 2.3 Cell Fabrication

The electrolyte‐supported SOFCs were fabricated by utilising the tape‐casting method combined with brush coating. The aqueous emulsion system was combined with the LSGM electrolyte powder in three stages and ball‐milled for 3 h per stage to ensure a homogeneous slurry. The detailed procedure for electrolyte slurry preparation has been reported in our previous study [35], with slight modifications. In particular, YSZ powder was replaced with LSGM powder, and thickening agents (TK004 and TK005) were added to obtain a viscous electrolyte slurry in order to produce a thick electrolyte layer. The slurry was then used to produce a 300‐micron LSGM electrolyte via tape‐casting onto a Teflon substrate through an Automatic Thick Film Coater (Shanyang Kejing Auto‐Instrument Co., Ltd.) and dried overnight at 40 °C. 30 mm‐diameter button cells were cut from the dried tape. These button cells were then sintered at 1450 °C for 4 h to obtain a dense, thick electrolyte support. LDC buffer layers were brush‐coated onto both sides of the dense LSGM electrolyte and then fired at 1250 °C for 2 h. The LSCF cathode is brush‐coated onto one LDC layer. Next, an anode layer with a weight ratio of HEO:GDC = 50:50 was brush‐coated onto the other LDC layer. Following the drying of the first HEO:GDC = 50:50 layer, a second anode layer having a weight ratio of HEO:GDC = 65:35, containing tapioca starch as a pore former, was then applied onto the first layer. These layers were then cosintered at 900 °C for 2 h, resulting in a complete electrolyte‐supported SOFC with the structure of HEO‐GDC(65:35)|HEO‐GDC (50:50)|LDC|LSGM|LDC|LSCF, as displayed in Figure 1.

**FIGURE 1:**
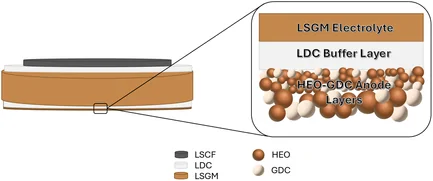
Electrolyte‐supported SOFC having cell architecture of HEO‐GDC(65:35)|HEO‐GDC (50:50)|LDC|LSGM|LDC|LSCF.

### 2.4 Material Characterization

The prepared HEO samples were systematically characterized by X‐ray diffraction (XRD), ICP‐OES, STEM‐EDX, SEM, XPS, Raman spectroscopy, and Micro‐CT to examine their structural and morphological features. Detailed experimental procedures for each characterization technique are provided in the Supporting Information. To further probe the local structure, Synchrotron‐based XAS analysis of the fresh d‐HEO(Fe) sample and respective standards was conducted at the XAS beamline of the Australian Synchrotron (ANSTO, Melbourne). The X‐ray absorption near edge structure (XANES) and extended X‐ray absorption fine structure (EXAFS) spectra were collected at room temperature in fluorescence mode at the Fe (7112 eV), Ni (8333 eV), Mn (6539 eV), Co (7709 eV), and Cu (8979 eV) K‐edges using a Si(111) double‐crystal monochromator. The calibration of the monochromator's energy was achieved by simultaneous measurement of Ni, Fe, Mn, Co, and Cu metal foils alongside the samples. Following the dilution in cellulose powder, the samples were pressed into pellets (*Ø* = 1.3 cm). The resultant pellets were encapsulated by Kapton tape and then placed on a sample holder for the measurement. The XANES and EXAFS data postprocessing were performed using ATHENA and ARTEMIS softwares, respectively [36].

### 2.5 Electrochemical Testing

The electrochemical performance of the LSGM electrolyte‐supported SOFCs was measured following the testing protocol reported in our previous works [35, 4]. A testing station—The Open Flanges test fixture (Fiaxell SOFC Technologies, Switzerland)—was employed. The current collection of the cathode was achieved by applying silver paste directly onto the cathode with 14 mm diameter, which was attached to silver mesh. This current collector was insulated from the test rig using alumina felt. The 20 mm diameter anode current collection was achieved through nickel foam. Mica sheet was utilized for the gas sealing between the anode and cathode sides, four calibrated springs applying equal load onto the testing manifold to ensure sealing. The electrolyte‐supported SOFC button cell was heated to 900 °C and left to reduce under 40 mL/min of humid hydrogen (3% H_2_O) over the anode for 24 h with 80 mL/min of Argon over the cathode to balance pressures across the cell. Next, 27 mL/min of anhydrous NH_3_ was fed to the anode, whereas compressed air was delivered to the cathode at a flow rate of 100 mL/min. The direct ammonia‐fed SOFC testing was conducted at various temperatures of 700, 750, 800, and 850 °C. Additionally, a durability test was conducted over 75 h at a constant current of 268 mA/cm^2^ and temperature of 800 °C at dry ammonia and air flowrates of 27 and 80 mL/min, respectively. Electrochemical impedance spectroscopy (EIS) spectrum was also collected, utilising Zahner IM6 electrochemical workstation, at a frequency range of 0.1–20 kHz under open circuit voltage (OCV) condition.

### 2.6 Computational Methodology

Quantum ESPRESSO package was used to carry out the DFT calculations, wherein the generalized gradient approximation (GGA) with the Perdew–Burke–Ernzerhof (PBE) exchange–correlation functional was employed [37]. The projector augmented wave (PAW) method was adopted to describe ion–electron interactions [38]. A plane‐wave kinetic energy cutoff of 50 and 30 Ry was considered for calculations involving the Fe(110) and Ni(111) slabs, respectively. Monkhorst–Pack (MP) mesh size of 2 × 2 × 1 was chosen, and a 20 Å vacuum layer was introduced to eliminate interactions between neighbouring images. Spin‐unrestricted calculation was conducted for calculations related to the Fe(110) surface to account for the ferromagnetic nature of the Fe atoms; however, nonpolarized calculation was adopted for calculations involving the Ni(111) surface. Structural relaxation computations converged when the forces on atoms and total energy are lower than 10^–3^ Ry/au and 10^–4^ Ry, respectively. The adsorption energy calculation details have been reported in previous studies [39, 40, 41]. The transition states of each reaction step, having seven images, were computed through climbing‐image nudged elastic band (CI‐NEB) method having force convergence threshold of 0.05 eV/Å. The equations adopted for calculating the forward (*E*_a,f_) and backward (*E*_a,b_) activation barriers as well as reaction energy (Δ*E*) were reported elsewhere [42, 43, 44, 45, 46]. Charge–transfer analysis was performed to obtain charge density difference (CDD) plots and to quantify the charges of the adsorbate using Bader charge analysis. As regards the computational models, the (111) and (110) surfaces were selected as the exposed facets of Ni and Fe slabs, respectively, as they represent the most stable surfaces of face‐centered cubic (fcc) Ni and body‐centered cubic (bcc) Fe [40, 47]. The Fe(110) and Ni(111) surfaces were modelled using a supercell size of 3 × 3 unit cells, each having five atomic layers (Figure S1).

## 3 Result and Discussion

### 3.1 Structural and Chemical Characterization

The XRD spectra of the fresh HEO powders were collected (Figure 2a), and their corresponding Rietveld refinement analyses are listed in Table S1. The HEO(Zn) and HEO(Mg) samples exhibited a single‐phase CuFe_2_O_4_ with a face‐centered cubic crystal structure (PDF: 04‐027‐8215, space group Fd‐3m). The diffraction peaks observed at 2*θ* = 30.18°, 35.55°, 37.19°, 43.22°, 53.63°, 57.15°, and 62.77° can be assigned to the (220), (311), (222), (400), (422), (511), and (440) planes, respectively [48, 49]. The splitting of the (222), (400), and (440) spinel reflections suggests significant structural distortion stemming from the incorporation of multivalent metal cations that disrupt the uniformity of metal–oxygen bonding within the HEO lattice [50]. The spinel‐structure CuFe_2_O_4_ has a lattice parameter a of 8.394 and 8.374 Å for the HEO(Zn) and HEO(Mg) samples, respectively (Table S1). On the contrary to the other samples, the d‐HEO(Fe) demonstrated a dual‐phase structure composed mainly of cubic CuFe_2_O_4_ (90.9 wt%) with a minor hematite phase (Fe_2_O_3_, 9.1 wt%, PDF 04‐005‐6837, space group: R‐3cH). The Fe_2_O_3_ phase has a hexagonal crystal structure (*a* = 5.034 Å, *c* = 13.746 Å).

**FIGURE 2:**
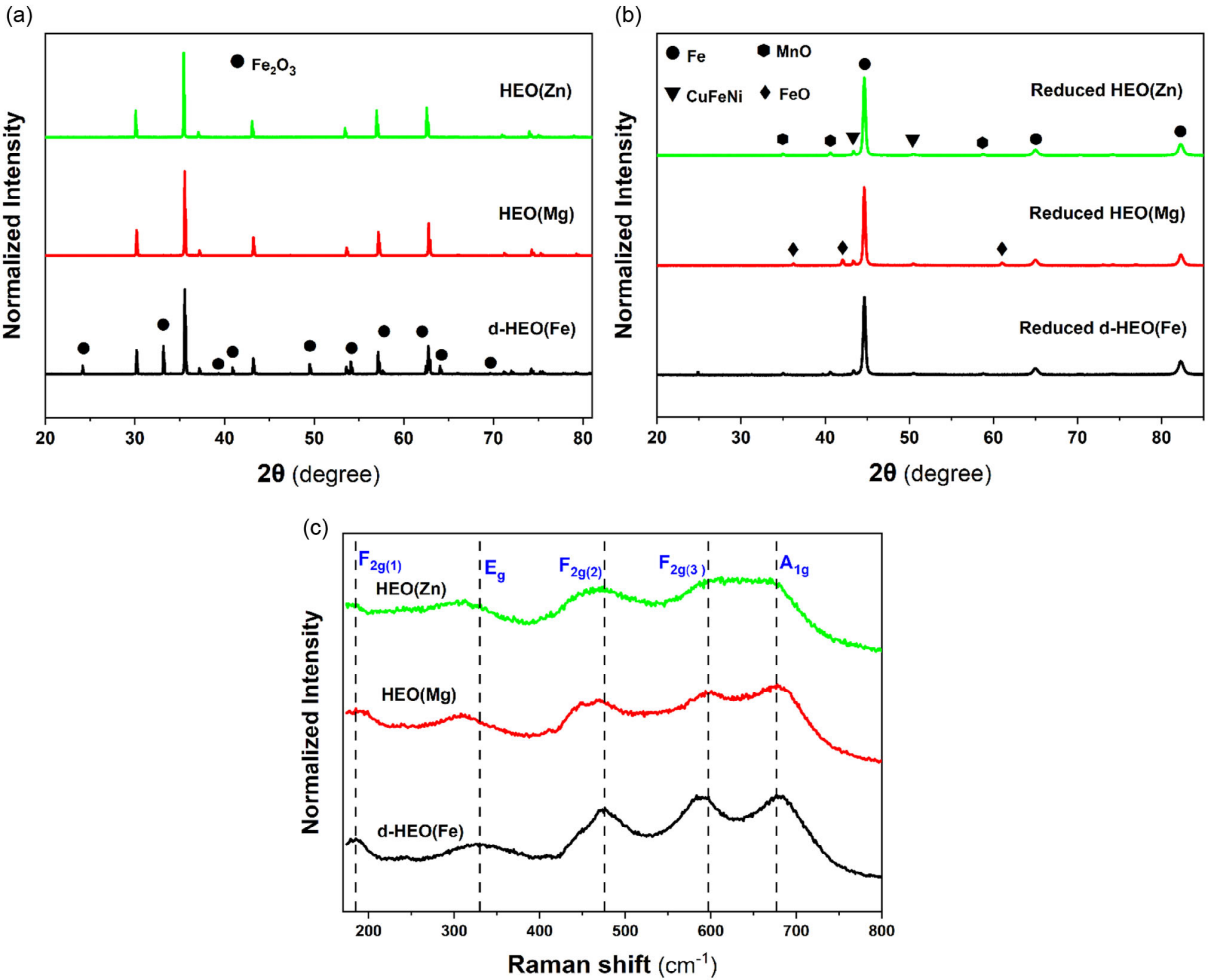
XRD spectra of the (a) fresh and (b) reduced d‐HEO(Fe), HEO(Zn), and HEO(Mg) samples, along with (c) Raman spectra of the corresponding fresh HEO samples.

Following exposure to 5% H_2_ in 95% N_2_ atmosphere at 900 °C for 16 h, the spinel oxide structure of all HEO samples was decomposed, yielding predominantly a monometallic Fe phase (PDF 04‐003‐3884, space group Im‐3m), along with a FeNiCu ternary alloy phase (PDF 04‐028‐0523, space group Fm‐3m) in varying proportions (Figure 2b). In addition, a secondary MnO phase (PDF 04‐002‐8159, space group Fm‐3m) was detected in the HEO(Zn) and d‐HEO(Fe) samples, whereas FeO phase (PDF 04‐004‐7638, space group Fm‐3m) was identified in the HEO(Mg) sample. Among the reduced samples, the d‐HEO(Fe) showed the highest Fe fraction at 88.9 wt%, while the HEO(Zn) and HEO(Mg) samples contained 88.0 and 82.4 wt% Fe, respectively (Table S2).

To explore the local structure, Raman spectra of the three HEO samples were collected in the wavenumber window of 175–800 cm^−1^ (Figure 2c). The position of Raman peaks and their vibrational assignments are listed in Table S3. The peak centered at 669–677 cm^−1^ can be attributed to *A*_1g_ originated from symmetric stretching of oxygen atoms bonded to tetrahedral cation (AO_4_) [51, 52]. The literature reported that the normal spinels have *A*_1g_ mode in the Raman spectral range of 600–620 cm^−1^, whereas this mode in inverse spinel ferrites occurs between 670 and 710 cm^−1^ [53]. Therefore, the occurrence of *A*_1g_ mode between 669 and 677 cm^−1^ indicates that the three HEO samples have a partially inverse spinel structure. Clearly, the position of the *A*_1g_ mode shifts from ~669 cm^−1^ for HEO(Zn) to higher wavenumbers (~677 cm^−1^) for d‐HEO(Fe) and HEO(Mg), as listed in Table S3. This blue shift is attributed to the substitution of heavier (65.37 amu) and larger Zn^2+^ ions (~0.60 Å) with lighter and smaller cations such as Mg^2+^ (24.31 amu, ~0.57 Å) and Fe^3+^ (55.85 amu, ~0.49 Å). The lighter mass and smaller ionic radii result in shorter and stronger M–O bonds at the tetrahedral sites, leading to an increase in vibrational frequency [53]. The Raman peak at around 600 cm^−1^ is indexed to *F*_2g(3)_ vibrational mode, arising from the asymmetric bending of metal–oxygen (M–O) bonds within the octahedral coordination environment [51, 52]. It can be noted that the HEO(Zn) sample demonstrated a very broad peak, extending from 613 to 669 cm^−1^, which is most probably due to a considerable structural disorder within the structure [54]. The *F*_2g(2)_ mode (470–476 cm^−1^) is relevant to asymmetric stretching at M–O bond in an octahedrally coordinated environment [51, 52]. Interestingly, the *F*_2g(2)_ peak exhibits an additional subpeak or shoulder in the three HEO samples, reflecting the distribution of cations at the octahedral sites. This peak splitting arises from variations in the force constant (k) of the cations within the lattice [55]. The *E*_g_ Raman band observed below 380 cm^−1^ is widely recognized as a distinctive feature of ferrite spinel structures [51]. Accordingly, the band that appeared between 308 and 330 cm^−1^ can be correlated with symmetric bending of oxygen atoms coordinated to metal cations. The *F*_2g(1)_ mode (181–185 cm^−1^) is ascribed to the translational vibrations of metal cations coupled with oxygen atoms at the tetrahedral sites (AO_4_) [51]. It should be noted that the intensity of the *F*_2g(3)_ Raman mode increases with the strengthening covalent character of the M—O bonds at the octahedral sites [56]. The peak intensity of *F*_2g(3)_ mode followed the order: d‐HEO(Fe) > HEO(Mg) > HEO(Zn), signalling that the d‐HEO(Fe) exhibits the strongest covalency of M—O bond among the investigated HEO samples.

The chemical compositions of the fresh HEO samples were quantitatively determined via ICP‐OES (Table S4). The ICP‐OES results on multiple samples indicated that the molar ratio of Ni:Cu:Co:Mn:Fe in d‐HEO(Fe) is 0.21:0.20:0.14:0.14:2.30, while the M:Ni:Cu:Co:Mn:Fe ratios are 0.28:0.20:0.20:0.13:0.13:2.00 for the HEO(Zn) and 0.26:0.20:0.20:0.13:0.13:2.00 for the HEO(Mg). These values closely match the stoichiometric ratios expected from the HEO chemical formula, indicating successful synthesis of high‐entropy spinel oxide samples.

XPS analysis of the reduced HEO samples is performed, with emphasis on the core‐level spectra of Fe 2*p*, Cu 2*p*, and Ni 2*p* (Figure 3a–c) because they are associated with the metallic phases formed during reduction. The spectra for Mn 2*p*, Mn 3*s*, and Mg 1*s* are also provided in Figure S2. The Zn 2*p* and Co 2*p* spectra are not presented due to the absence of distinguishable peaks, suggesting concentrations below the XPS detection limit and/or subsurface localization or diffusion into the bulk. The XPS spectra of Fe 2*p* region of the three samples showed Fe^0^ peak, whose centroid is located at binding energy of 706.8 eV, signalling the existence of metallic Fe [57], which is consistent with XRD results. Furthermore, it can be noted that the relative Fe^0^ peak area followed the order d‐HEO(Fe) > HEO(Zn) > HEO(Mg), which is in excellent agreement with the Fe phase fraction trend derived from XRD. The Cu 2*p* and Ni 2*p*core level XPS spectra have peaks centered at 932.4 and 852.7 eV, respectively, corresponding to metallic Ni and Cu phases [57, 58], respectively. This observation corroborates the XRD findings, which evidence the formation of a ternary Fe–Cu–Ni alloy phase. Deconvolution of the Mn 2*p* spectra (Figure S2a) reveals the presence of Mn^2+^ and Mn^4+^ species in d‐HEO(Fe) and HEO(Zn), while Mn^2+^ and Mn^3+^ are identified in HEO(Mg), confirming that manganese exists in mixed‐valence states across all reduced samples [59, 60, 61, 62]. The Mn 3*s* XPS spectrum (Figure S2b) exhibits characteristic multiplet splitting, with an energy separation Δ*E*_Mn3s_ of 5.4–5.7 eV, indicative of the presence of Mn^2+^ species [63]. The Mg 1*s* core‐level XPS spectrum of the HEO(Mg) sample shows a distinct peak at 1303.3 eV, corresponding to metallic Mg, which is not detected by XRD (Figure S2c).

**FIGURE 3:**
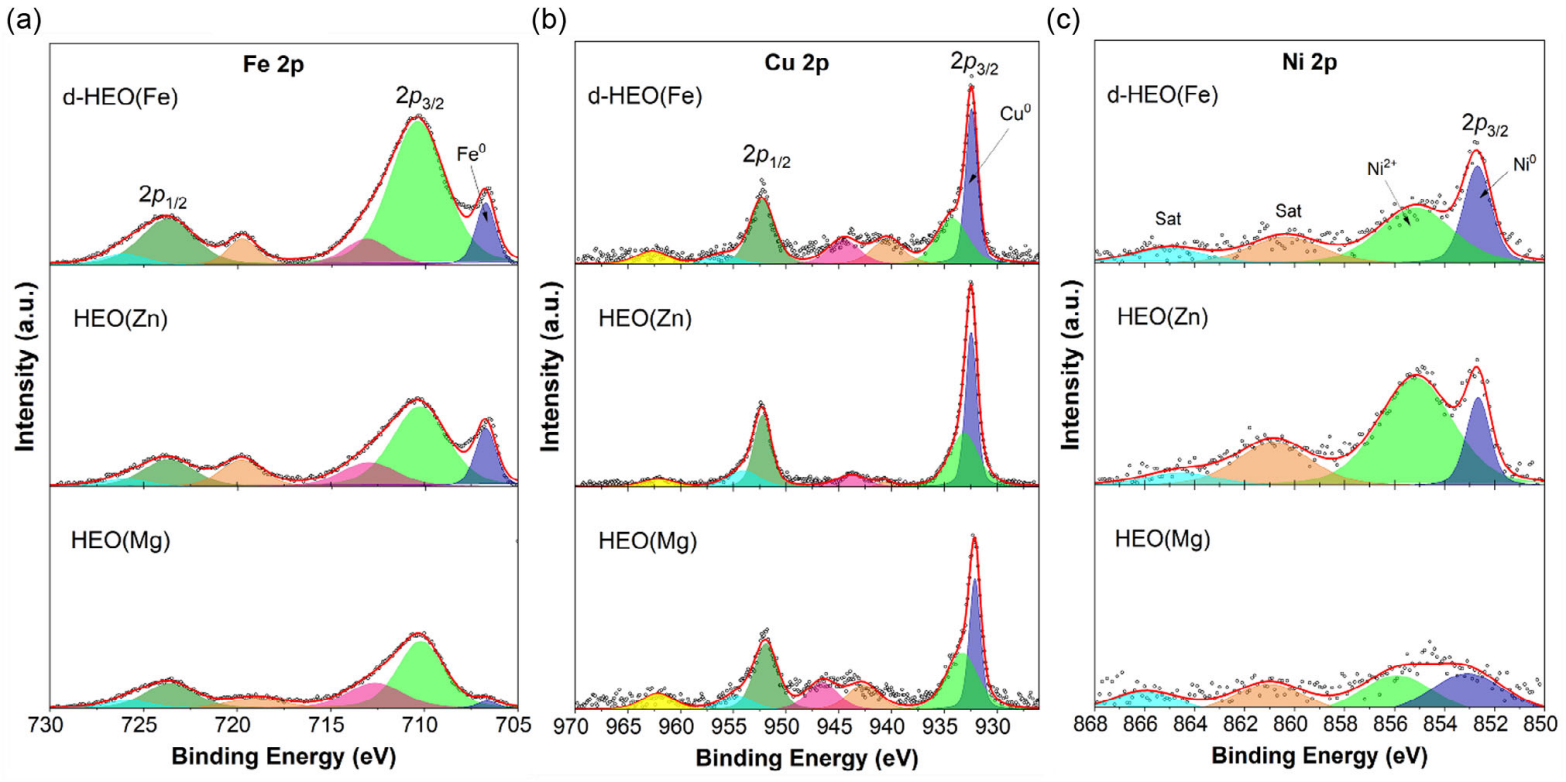
XPS spectra of reduced HEO samples; (a) Fe 2p, (b) Cu 2p, and (c) Ni 2p.

### 3.2 XAS Insights Into Local Environment, Electronic Structure, and Cation Occupancy

XANES analysis provides qualitative insight into the average electronic state of the absorbing metal by evaluating the edge energy relative to reference compounds with well‐defined oxidation states. The XANES spectra of the fresh d‐HEO(Fe) sample acquired at the Fe, Cu, Ni, Co, and Mn K‐edges are displayed in Figure 4a–e, alongside the references (Fe, Fe_2_O_3_, FeO, Cu, Cu_2_O, CuO, Ni, NiO, Co, Co_3_O_4_, CoO, Mn, MnO, and MnO_2_). Additionally, the absorption energies and electronic states of the d‐HEO(Fe) sample at different K‐edges as well as standards are listed in Table 1. The edge energy of Fe K‐edge XANES spectrum of the d‐HEO(Fe) sample is similar to the Fe_2_O_3_ standard, signalling that the Fe(III) is the dominant electronic state for Fe element in the sample. Additionally, the d‐HEO(Fe) exhibited an intense pre‐edge peak, which is a characteristic of Fe^3+^ ions located at tetrahedrally coordinated environments [64]. The absorption energy of the Cu, Ni, and Co K‐edges of the d‐HEO(Fe) sample is comparable to those of the respective metal oxides (MO, M = Cu, Ni, and Co), highlighting the existence of Cu^2+^, Ni^2+^ and Co^2+^ ions in the sample. On the other hand, the Mn K‐edge energy of the sample is higher than that of MnO and lower than that of MnO_2_ while closely matching that of Mn_2_O_3_ [65, 66], suggesting the presence of Mn(III) species in the sample.

**FIGURE 4:**
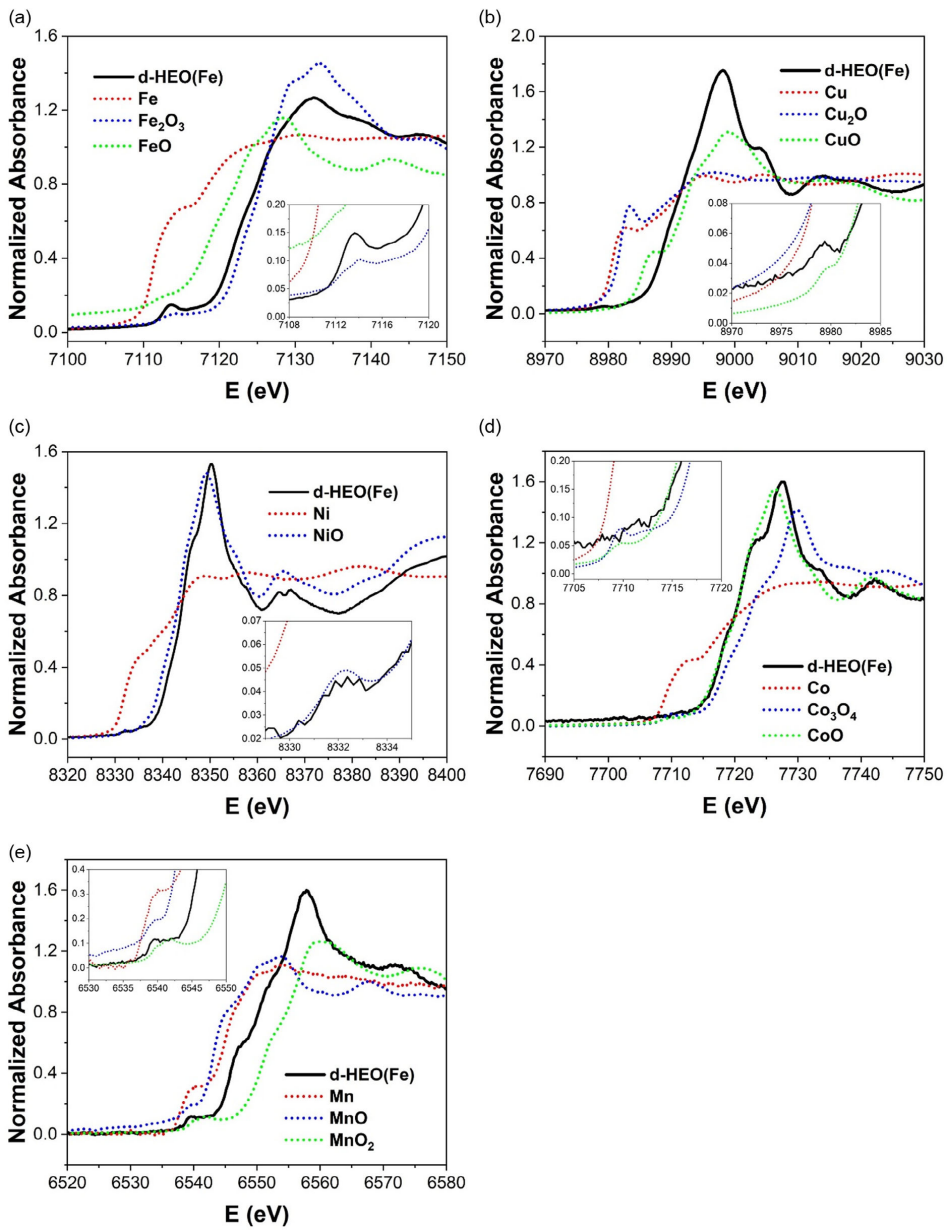
XANES spectra of the fresh d‐HEO(Fe) sample acquired at the (a) Fe, (b) Cu, (c) Ni, (d) Co, and (e) Mn K‐edges, alongside the Fe, Fe_2_O_3_, FeO, Cu, Cu_2_O, CuO, Ni, NiO, Co, Co_3_O_4_, CoO, Mn, MnO, and MnO_2_ standards.

**TABLE 1:** Edge energy and valence states for the d‐HEO(Fe) sample along with the standards.

Sample	Element	Oxidation state	*E*_o_, eV
d‐HEO(Fe)	Fe	+3	7125.4
	Cu	+2	8995.4
	Ni	+2	8344.7
	Co	+2	7721.5
	Mn	+3	6545.6
Fe_2_O_3_	Fe	+3	7125.7
FeO		+2	7122.8
Fe		0	7111.1
CuO	Cu	+2	8992.6
Cu_2_O		+1	8982.1
Cu		0	8980.6
NiO	Ni	+2	8343.9
Ni		0	8331.9
Co_3_O_4_	Co	+2, +3	7728.1
CoO		+2	7721.1
Co		0	7708.8
MnO_2_	Mn	+4	6556.9
MnO		+2	6542.9
Mn		0	6538.8

Qualitative analysis of Fourier transform (FT) of k^3^χ(k) EXAFS spectra has been widely used as a fingerprinting tool to identify the site occupancy of metal cations in high‐entropy spinel structures [67, 68]. The EXAFS spectra of the fresh d‐HEO(Fe) sample, alongside the corresponding metal foil, at Fe, Cu, Ni, Co, and Mn K‐edges are presented (Figure 5a–e). In spinel crystal structure, there are two cationic sites: 16d site in the octahedral coordination and 8a site in tetrahedral coordination [69]. In EXAFS spectra, the first peak, ascribed to metal–oxygen bond (M–O), is located at about 1.5 Å [70]. This M–O peak was observed in the EXAFS spectra across all studied metal species (Fe, Cu, Ni, Co, and Mn). The coordination environment of cations in spinel structures can be resolved in the 2.2–3.5 Å R‐region, with octahedrally coordinated sites characterized by peak at lower R values and tetrahedrally coordinated sites exhibiting a single peak at higher *R* values [69, 68]. The Fe K‐edge EXAFS spectrum reveals a strong peak at 2.7 Å and a weaker peak at 3.3 Å, suggesting that Fe^3+^ cations are mainly located in octahedral sites, while a minor fraction occupies tetrahedral coordination environments. Moreover, an additional low‐intensity peak at 2.3 Å was noticed, which is consistent with that of the metallic Fe foil, evidencing the existence of Fe^0^ particles that are not detectable by XRD. This discrepancy arises because XRD detects only long‐range crystalline order, requiring sufficiently large Fe domains, whereas EXAFS probes local Fe–Fe coordination and can identify metallic Fe even when it is highly dispersed or nanosized. The Cu and Ni showed a high‐intensity peak at 2.6 Å, alongside weak peak in the R region of 3.0–3.1 Å, indicating that the Cu^2+^ and Ni^2+^ cations are distributed between octahedral and tetrahedral coordination sites. Notably, the Ni K–edge demonstrated an additional peak at 2.2 Å, which closely matches that of the Ni foil, corresponding to the characteristic Ni–Ni bond. Similarly, the Co K‐edge EXAFS shows a prominent peak at ≈2.6 Å and a shoulder near 3.2 Å, suggesting that the majority of Co^2+^ cations are located in octahedral coordination sites, with the remaining fraction occupying tetrahedral positions. On the other hand, a very intense peak was observed for the Mn K‐edge at about 2.6 Å, implying that the Mn^3+^ cations are octahedrally coordinated in the spinel. It can be inferred that the distribution of Fe^3+^, Ni^2+^, Cu^2+^, and Co^2+^ cations between the tetrahedral and octahedral coordination environments with the spinel suggests that the fresh d‐HEO(Fe) sample has a partially inverse spinel structure, which coincides well with the Raman results.

**FIGURE 5:**
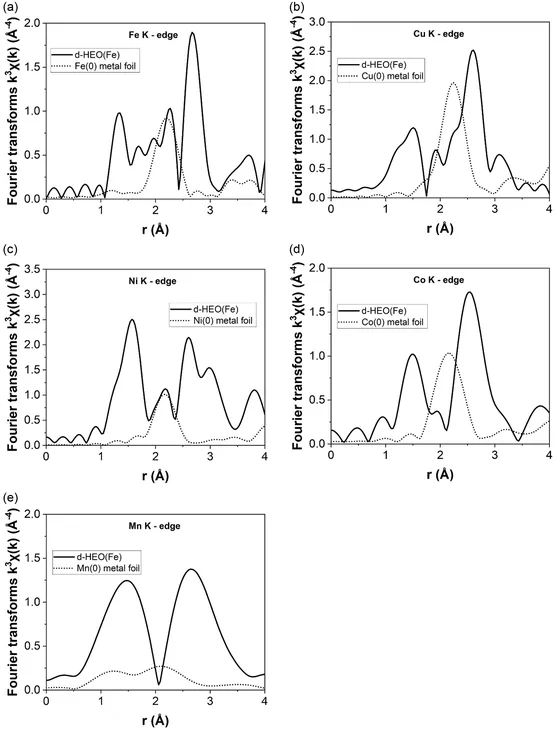
Fourier transform of k^3^χ(k) EXAFS spectra of the fresh d‐HEO(Fe) sample, together with the corresponding metal foil, at (a) Fe, (b) Cu, (c) Ni, (d) Co, and (e) Mn K‐edges. The EXAFS signals of the Ni and Co foils are scaled by a factor of 1/3, while that of the Fe foil is scaled by 1/2.

### 3.3 Electron Microscopy Analyses

Scanning electron micrographs of the fresh and reduced HEO samples along with the half‐cell cross‐section are depicted in Figure 6a–g. All fresh samples showed a dense and well‐defined morphology with smooth surfaces, indicative of highly crystalline oxide phases. While particles in the fresh d‐HEO(Fe) sample displayed well‐defined terrace‐like morphology, the fresh HEO (Zn) sample possessed highly uniform polyhedral particles with sharp edges and flat faces. The fresh HEO (Zn) had compact morphology with irregular particle shapes. The three reduced samples underwent pronounced morphological modification, manifested by irregular morphology accompanied by increased surface roughening, suggesting partial structural collapse and lattice reconstruction driven by oxygen removal and cations rearrangement during reduction process, which is consistent with the phase transformation revealed by XRD results. The STEM‐EDX mapping of the reduced d‐HEO(Fe) sample (Figure 7) revealed the uniform distribution of Fe, Cu, Ni, Co, and Mn metals across the whole region.

**FIGURE 6:**
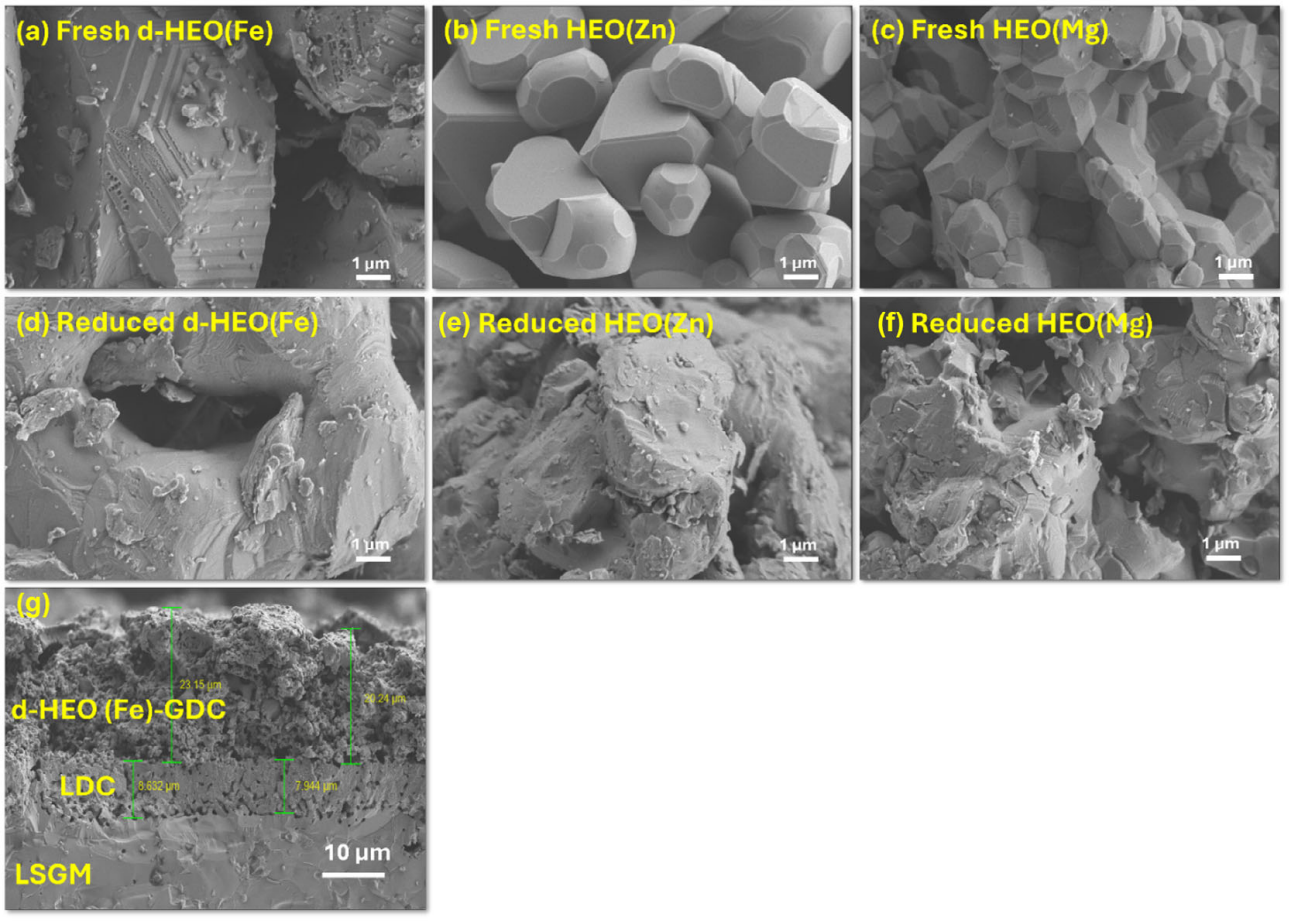
SEM images of (a–c) fresh and (d–f) reduced d‐HEO(Fe), HEO(Zn), and HEO(Mg) samples, alongside (g) the cross‐section of the half‐cell having d‐HEO(Fe)‐GDC anode.

**FIGURE 7:**
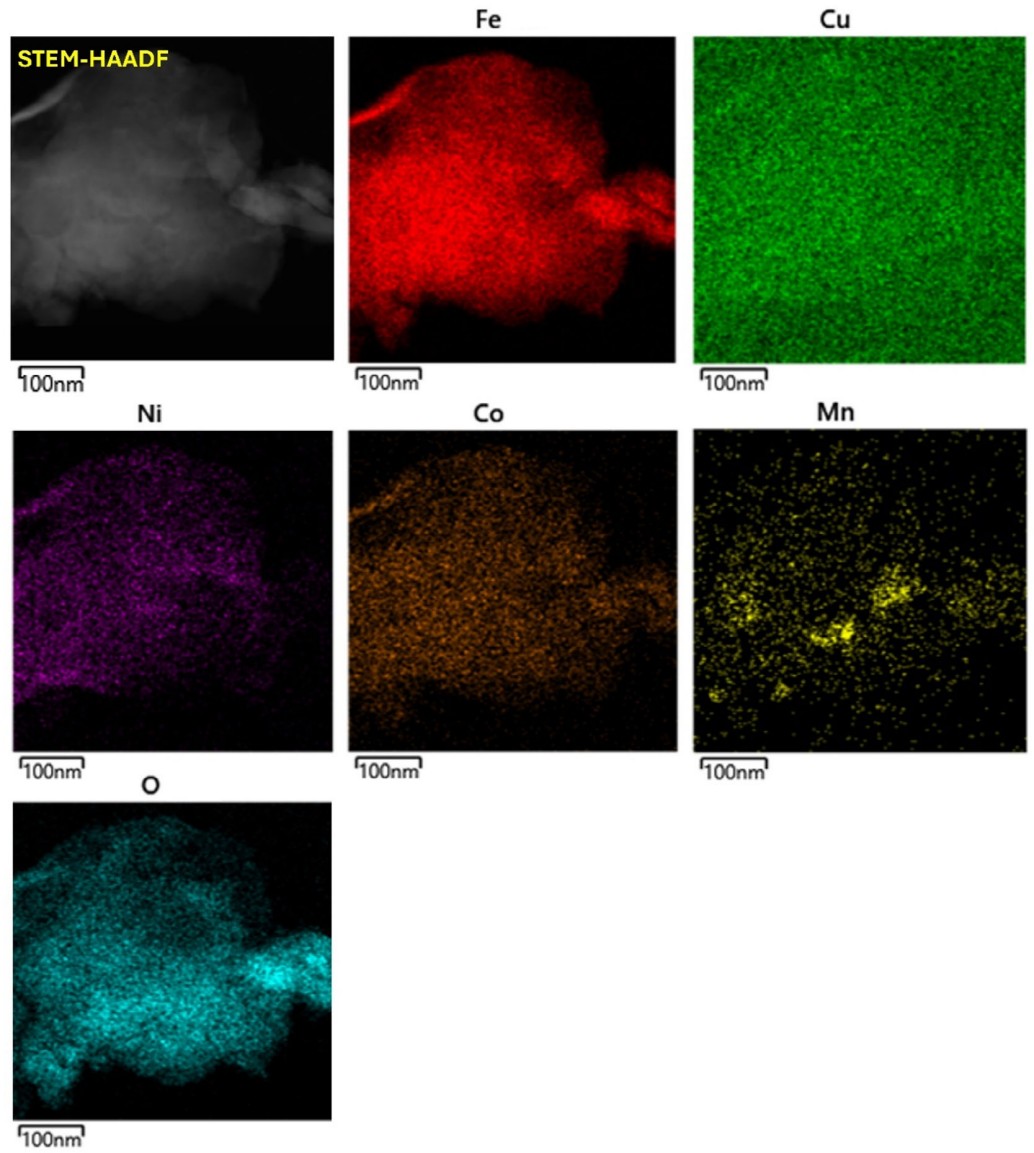
STEM‐EDX image of the reduced d‐HEO(Fe) sample.

### 3.4 3D Microstructural Quantification

The 3D grayscale and segmented reconstructions of the reduced samples, highlighting the Fe, FeNiCu, MO (M = Mn or Fe), GDC, and pore phases, are presented (Figure 8a–f). The raw and segmented 2D slices of the three reduced anodes are displayed in Figure S3. It can be observed that the d‐HEO(Fe) anode demonstrates a greater extent of Fe‐phase percolation relative to the other anodes, which can be ascribed to its increased Fe content. The volume fraction of each phase and corresponding local fractions across the *Z*‐direction towards the anode/electrolyte interface were quantified (Figure 9a). The Fe phase is the most predominant constituent in the three anodes. Notably, the Fe content of the three anodes decreases in the order d‐HEO(Fe) (62%) > HEO(Zn) (61%) > HEO(Mg) (58%), which is in good agreement with the Fe phase fractions obtained from XRD measurements of the corresponding reduced powders. The high Fe content is beneficial for the electrochemical performance as it enhances both ammonia decomposition, as will be elaborated upon latter in DFT analysis section, alongside the electrochemical oxidation of hydrogen [71]. The volume fraction of GDC phase (22%–23%) is almost identical for the three anodes, consistent with the nominal loading (20 wt%) during the fabrication process. Interestingly, no significant variations are observed in the local volume fractions of the Fe, FeNiCu, MO (M = Mn or Fe), GDC, and pore phases along the *Z*‐direction for all three anodes (Figure 9b–f), indicating good microstructural homogeneity.

**FIGURE 8:**
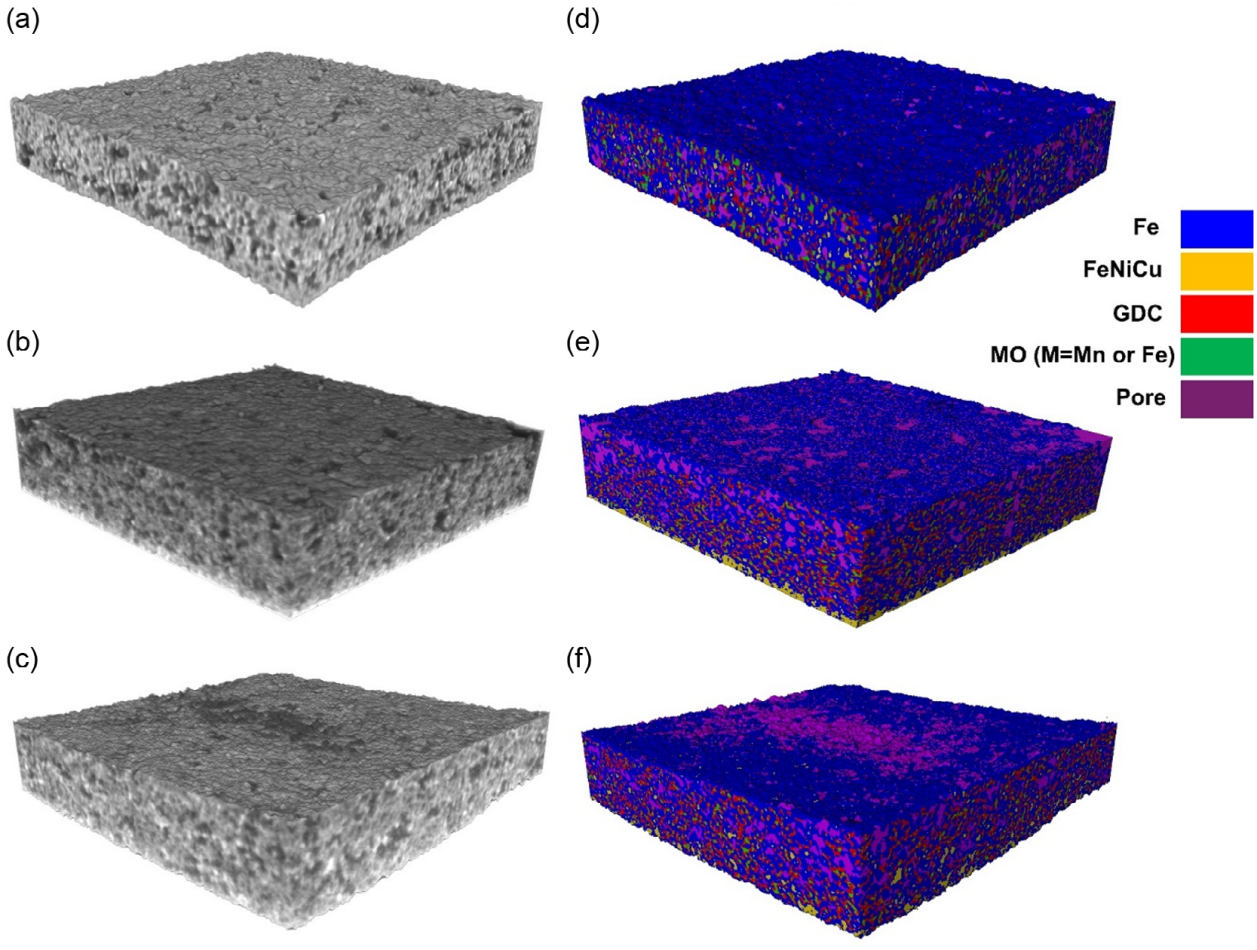
3D grayscale and segmented reconstruction of the microstructure of reduced d‐HEO(Fe) (a,d) and HEO(Zn) and (b,e) HEO(Fe) (c,f) samples.

**FIGURE 9:**
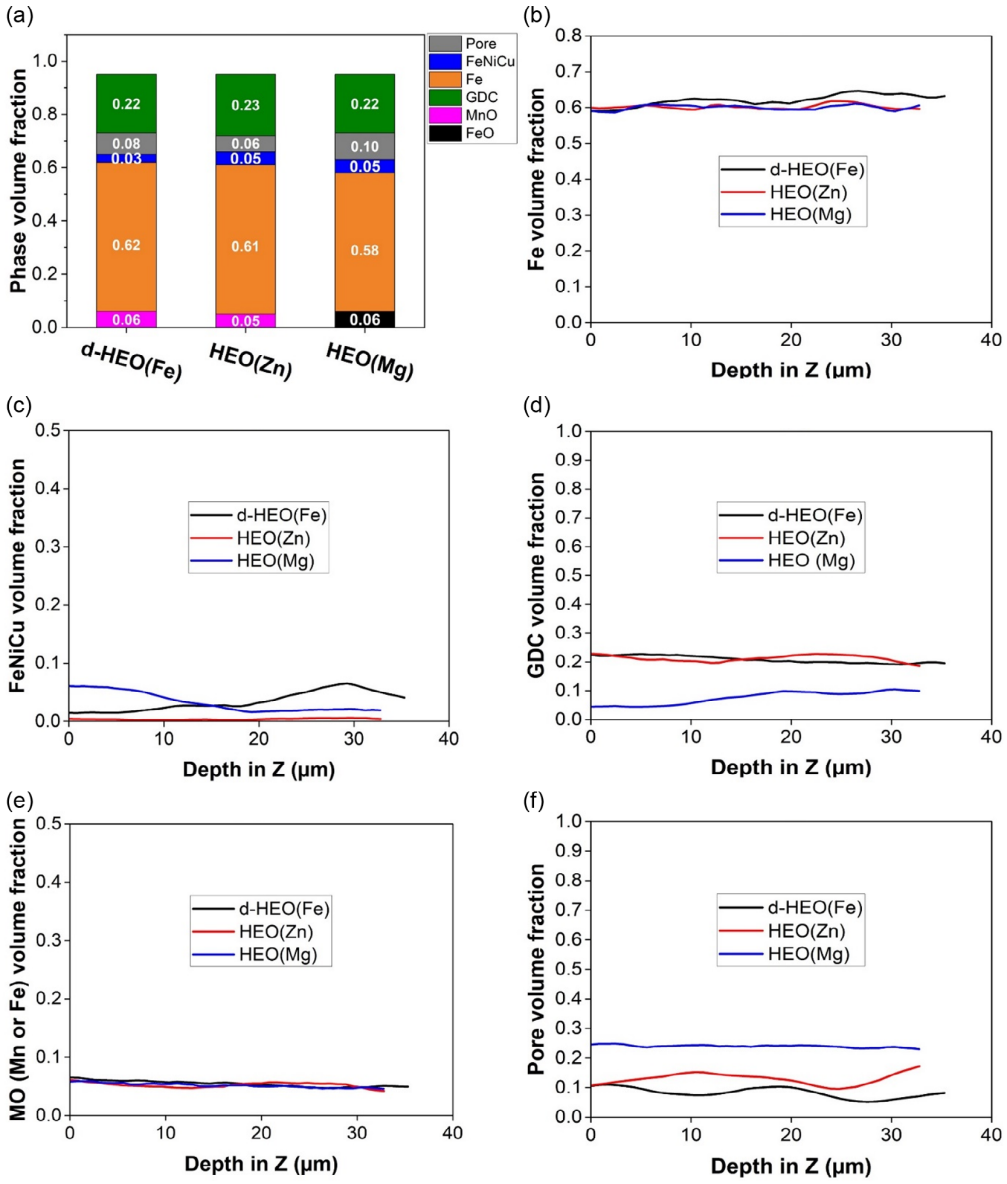
(a) Phase volume fraction, local volume fraction as a distance across the *Z*‐direction for (b) Fe, (c) FeNiCu, (d) GDC, (e) MO (M = Mn or Fe), and (f) pore phases along the *Z*‐direction of the reduced HEO samples.

The quantified specific surface area (SSA) and interfacial area (SIA) per unit volume of each constituent phase for the reduced anodes are presented (Figure 10a,b). Notably, the Fe phase exhibits the highest specific surface area across all anodes due to its higher volume fraction relative to other phases. The SSA values follow the order: d‐HEO(Fe) > HEO(Mg) > HEO(Zn). Notably, the HEO(Zn) anode exhibits the lowest SSA despite having a higher Fe content than the HEO(Mg) counterpart, suggesting significant coarsening of Fe particles. Among all phase–phase pairs, the Fe and GDC phases share the largest interfacial contact area in the three anodes, while Fe–pore pair also has relatively high SIA values. This dominance of Fe–GDC interfacial contact, together with the substantial Fe–pore interfacial area, is highly favourable for electrochemical performance as it enhances the density of electrochemically active triple phase boundary regions by coupling electronic conduction (Fe) and ionic conduction (GDC), along with gas transport (pores). Furthermore, the local Fe–GDC‐specific interfacial contact density is nearly uniform along the *Z*‐direction for all anodes (Figure 10c), indicating a homogeneous distribution of electrochemically active interfaces through the anode thickness. The intersection of the Fe, GDC, and pore phases, providing electronic conduction, ionic conduction and gas diffusion pathways, respectively, is anticipated to be the three‐phase boundary region—analogous to the Ni–YSZ–pore TPBs in conventional Ni–YSZ anodes [35]—which is the active site for the hydrogen electrochemical oxidation reaction. Accordingly, the TPB densities for the reduced HEO anodes are estimated (Figure 10d). The TPB density, obtained from micro‐CT analysis, of the three anodes followed the sequence HEO(Mg) > d‐HEO(Fe) > HEO(Zn), with respective values of 0.083, 0.076, and 0.065 µm^−2^. The Feret diameter–based size distributions of Fe and GDC particles, together with pore size distribution, were also measured, as shown in Figure 11a–c. Clearly, the Fe grains and pores are mainly distributed within the size range of 1–4 µm for all anodes, with the majority of particles exhibiting a characteristic size of ≈1.25 µm. It is noteworthy that the d‐HEO(Fe) anode has the lowest fraction of larger particles (>1.25 µm) relative to other anodes. This observation confirms the possibility of pronounced coarsening of Fe particles within the HEO(Zn)‐ and HEO(Mg)‐based anodes, in accordance with the previously observed surface area trend of the Fe phase. The GDC particles in the d‐HEO(Fe) anode remained persistently larger and more frequent relative to those observed in the other two anodes.

**FIGURE 10:**
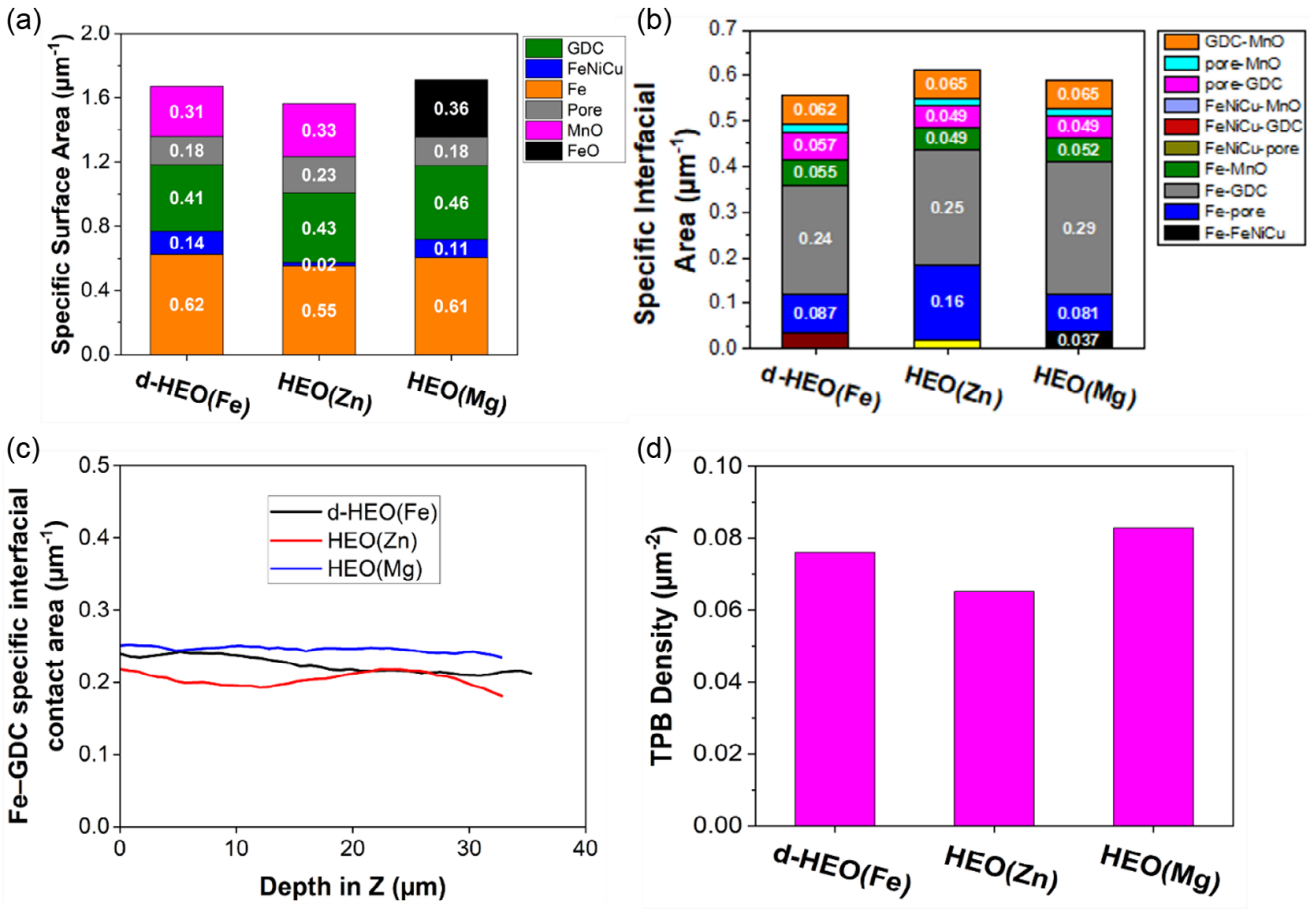
(a) Surface area per unit volume of each constituent phase; (b) interfacial area density between phases; (c) local Fe–GDC interfacial contact density; and (d) Fe–GDC–pore TPB density for the reduced samples.

**FIGURE 11:**
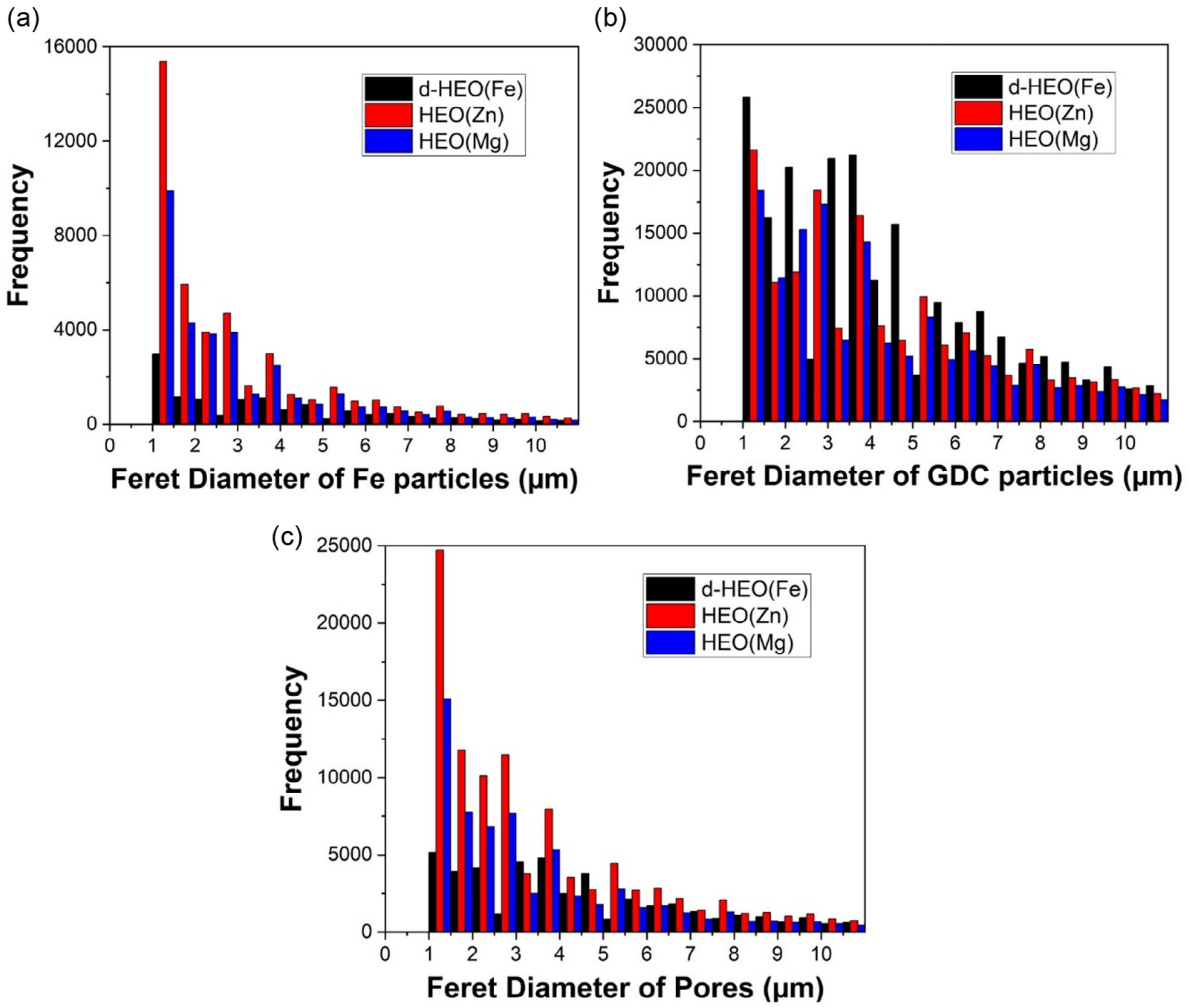
Feret diameter‐based size distributions of (a) Fe and (b) GDC particles, together with (c) pore size distribution.

### 3.5 Electrochemical Performance

The impedance spectra of the three cells are acquired under OCV condition at temperature range of 700–850 °C (Figure 12a–d). The resistance contribution at high frequencies is related to charge–transfer kinetics [72, 73, 74, 75], while the low‐frequency resistance is ascribed to fuel adsorption, conversion, and diffusion processes [73, 76, 77]. Over the entire temperature regime, the SOFC using d‐HEO(Fe)‐based anode consistently displayed the least ohmic resistance (∼0.47 Ω cm^2^ at 850 °C), determined from the *X*‐axis intercept of the Nyquist plot at the high frequency, compared to the cells having the HEO(Zn) (∼0.55 Ω cm^2^), and HEO(Mg) (∼0.59 Ω cm^2^) anodes. This is because of d‐HEO(Fe) anode has the greatest proportion of Fe phase upon reduction, which is known for its high electronic conductivity [78]. The polarization resistance, inferred from the diameter of impedance arc, is also the lowest when the d‐HEO(Fe) is employed as anode relative to the HEO(Zn) and HEO(Mg) contenders. The pronounced low‐frequency resistance contribution compared to the charge–transfer resistance, as observed in the EIS spectra, for the three cells indicates that gas diffusion constitutes a considerable transport limitation. As expected, a considerable increase in polarization resistance was observed for the three cells with decreasing temperature from 850 to 700 °C, exhibiting a thermally activated behaviour. This is probably arising from a diminished catalytic activity of the HEO anodes toward ammonia decomposition and H_2_ oxidation at lower operating temperatures.

**FIGURE 12:**
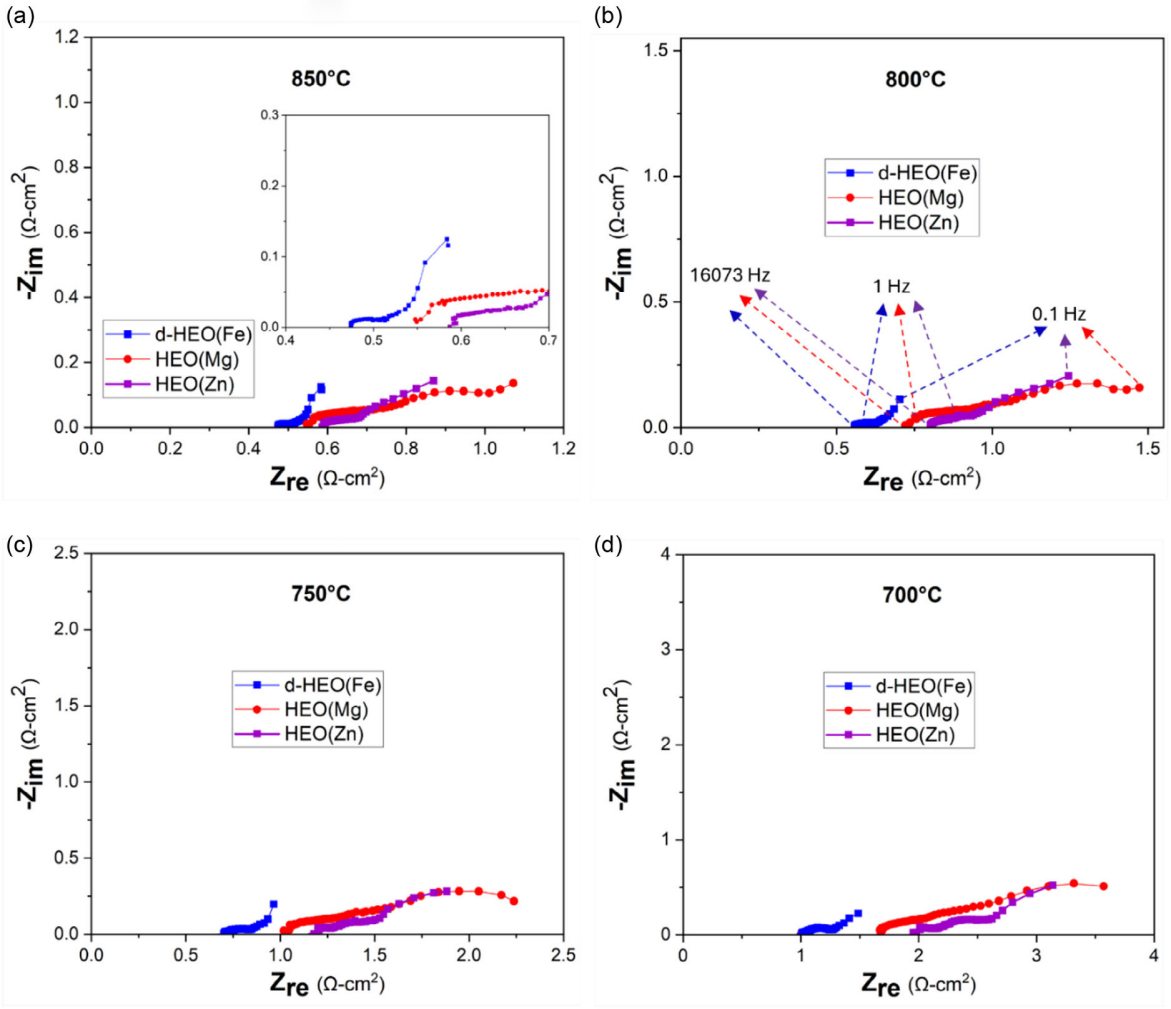
(a–d) EIS curves of the cells with d‐HEO(Fe), HEO(Zn), and HEO(Mg) anodes at 700–850 °C.

The electrochemical performance, in terms of current–voltage–power density curve, of the three SOFCs is examined under an ammonia atmosphere at 700, 750, 800, and 850 °C (Figure 13a–d). Apparently, the cell having d‐HEO(Fe) anode achieved the highest peak power density (PPD) of nearly 382.0 mW/cm^2^ at 850 °C, aligning with the EIS results previously described, whereas the SOFCs encompassing HEO(Zn) and HEO(Mg) anodes recorded lower PPD values of 218.3 and 195.7 mW/cm^2^, respectively. The superior electrochemical performance of the d‐HEO(Fe) anode arises from the synergistic interplay between efficient ammonia cracking and minimized ohmic resistance. The increased Fe phase content, confirmed by XRD results, promotes ammonia decomposition due to the high catalytic activity of Fe [79, 80, 81], while its high electronic conductivity results in the lowest ohmic resistance. It is noteworthy that, although the Fe–GDC–pore triple‐phase boundary regions are expected to serve as active sites for the H_2_ electro‐oxidation reaction at the anode, no direct correlation is observed between the electrochemical performance and the Fe–GDC–pore TPB density values obtained from micro‐CT analysis for the three anodes. On the other hand, the inferior electrochemical performance of HEO(Zn)‐ and HEO(Mg)‐based anodes can be correlated to the considerable coarsening of Fe particles, as evidenced by micro‐CT results, since it reduces the number of Fe active sites available for reaction. The cells presented a diminishing trend in the PPD values with temperature decrease from 850 to 700 °C, which could be correlated with increased ohmic resistance, originated from reduced LSGM ionic conductivity, and elevated polarization resistance, arising from suppressed NH_3_ decomposition rate and consequently lower hydrogen partial pressure, as evidenced by EIS results.

**FIGURE 13:**
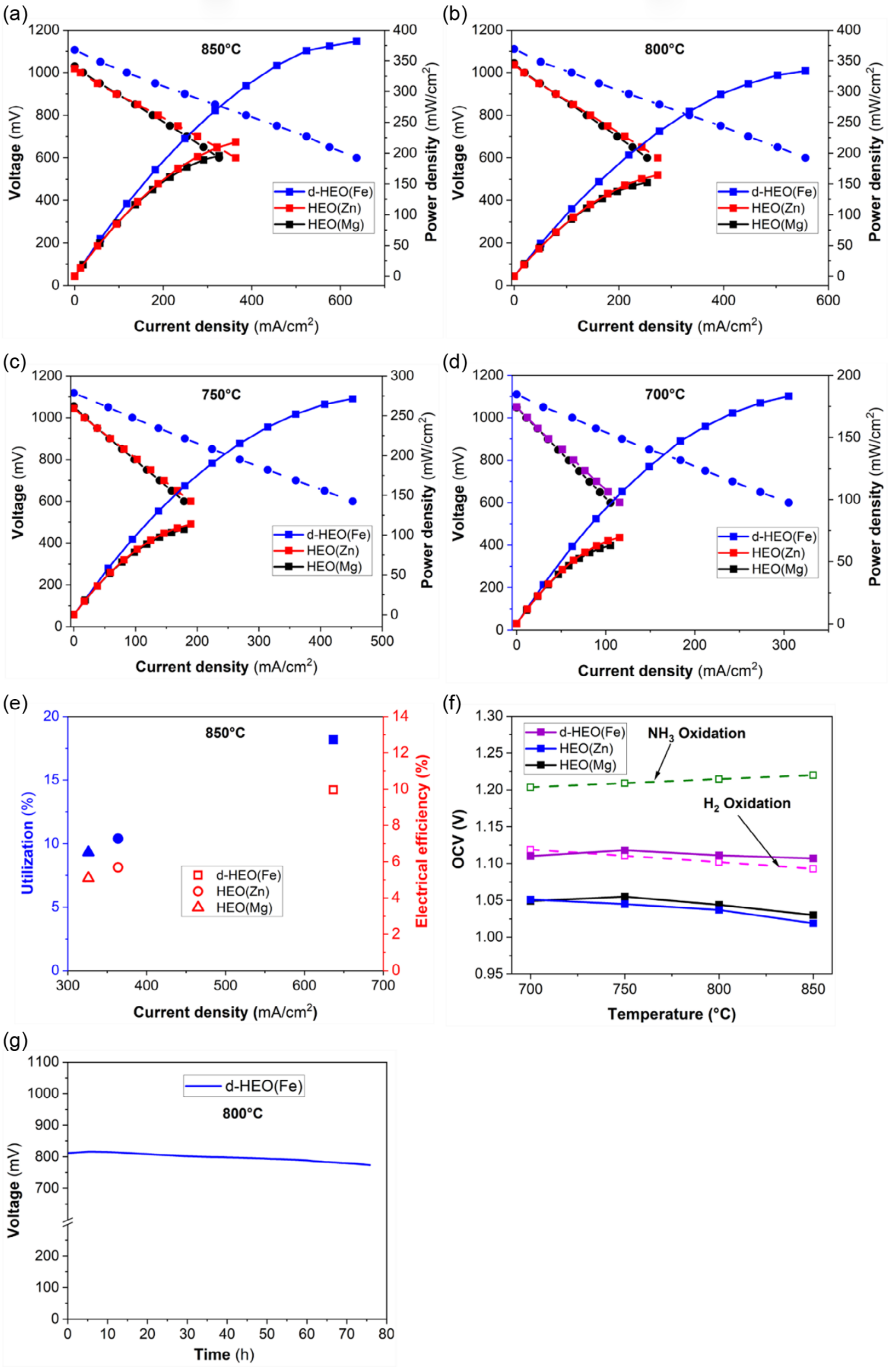
(a–d) Current–voltage–power density performance curves of the SOFCs incorporating the d‐HEO(Fe), HEO(Zn), and HEO(Mg)‐based anodes at temperature range of 700–850 °C. (e) Electrical efficiency and NH_3_ utilization at 850 °C, (f) experimental and theoretical OCV versus operating temperature for the three cells. (g) Stability test of the cell possessing d‐HEO(Fe) anode for 75 h under a current density load of 268 mA/cm^2^ at temperature of 800 °C.

Clearly, the cell containing the d‐HEO(Fe) anode exhibited the greatest fuel utilization and electrical efficiency of 18.2% and 10.0%, respectively, at 850 °C, whereas the cells having HEO(Zn) and HEO(Mg) anodes displayed inferior performance, with fuel utilization of 10.4% and 9.3% and electrical efficiency of 5.7% and 5.1%, respectively (Figure 13e). The experimental and theoretical OCV values were compared to discern the dominant reaction route of ammonia utilization on the different anodes (Figure 13f). The d‐HEO(Fe) and HEO(Mg) recorded the experimental OCV trend matches the trend of theoretical OCV trend for direct pathway of NH_3_ electro‐oxidation reaction at temperature range of 700*–*750 °C. However, at an elevated temperature range of 750–850 °C, the trend indicates the prevalence of the indirect pathway encompassing NH_3_ decomposition followed by H_2_ electrochemical oxidation. This OCV trend across the entire operating temperate range (700*–*850 °C) implies the occurrence of both direct and indirect pathways of ammonia utilization on these anodes, highlighting a combined reaction mechanism at play. Conversely, the cell with HEO(Zn) anode showed a diminishing trend of experimental OCV with temperature rise from 700 to 850 °C, signalling the dominance of the indirect route of ammonia utilization. Notably, the cell having d‐HEO(Fe) anode demonstrated a reasonable durability for 75 h at 800 °C (Figure 13g), exhibiting a low voltage degradation rate of only 3.16%, which might be associated with possible coarsening of Fe particles upon long‐term operation.

### 3.6 DFT‐Based Theoretical Investigation of NH_3_ Adsorption and Cracking Mechanism

#### 3.6.1 NH_3_ Adsorption

Given that the Fe metal is the dominant phase formed after the reduction‐driven decomposition of all HEO anodes, the ammonia adsorption and decomposition reaction mechanism was scrutinized on the Fe(110) surface and benchmarked against the Ni(111) slab. The ammonia adsorption at Fe atop site on the Fe(110) and Ni atop site on the Ni(111) surfaces is displayed (Figure 14a–d). The computed adsorption energy (*E*_ads_), mean N–H bond length (*d*_(N–H)_) of NH_3_ molecule_,_ and the nearest distance between the adsorbate and the substrate (*d*_(surf–ads)_) are tabulated (Table 2). The N–H distance of the gas‐phase NH_3_ molecule is determined to be 1.027 Å, in accordance with the experimentally measured value of 1.02 Å [82]. The NH_3_ adsorbs perpendicularly at the Fe atop site of Fe(110) surface. This NH_3_ adsorption configuration recorded the strongest adsorption energy of −1.20 eV, wherein NH_3_ is coordinated to the surface with its N atom, and the H atoms point outward. This strong adsorption has resulted in considerable structural deformation in the NH_3_ adsorbate, wherein the N–H bond experienced contraction to 1.023 Å in comparison to the isolated NH_3_ molecule. The adsorbate is relaxed at a height of 2.110 Å above the substrate. On the other hand, NH_3_ adsorbs less favourably at the Ni atop position of Ni(111) slab, through the N atom, with a weaker *E*_ads_ value of −1.04 eV relative to Fe atop site. Consistent with the weak adsorption, no structural deformation was noticed in the NH_3_ adsorbate, as evidenced by the *d*_(N–H)_ value being identical to that of the gas‐phase NH_3_ molecule. It should be noted that, during geometry optimization, adsorption configurations initially NH_3_ positioned at the Fe–Fe bridge and Fe hollow sites on the Fe(110) have eventually converged to the Fe atop site. A similar behaviour was observed on the Ni(111), wherein the NH_3_ is initially placed at the Ni–Ni bridge site; however, it migrated to the Ni atop position following structure relaxation. Overall, these results indicate that the Fe(110) surface enhances the adsorption strength of NH_3_ compared to the Ni(111) surface, which is in line with a previous study [80]. The two adsorption configurations were adopted as the initial states for studying the NH_3_ decomposition reaction on the Fe(110) and Ni(111) surfaces.

**FIGURE 14:**
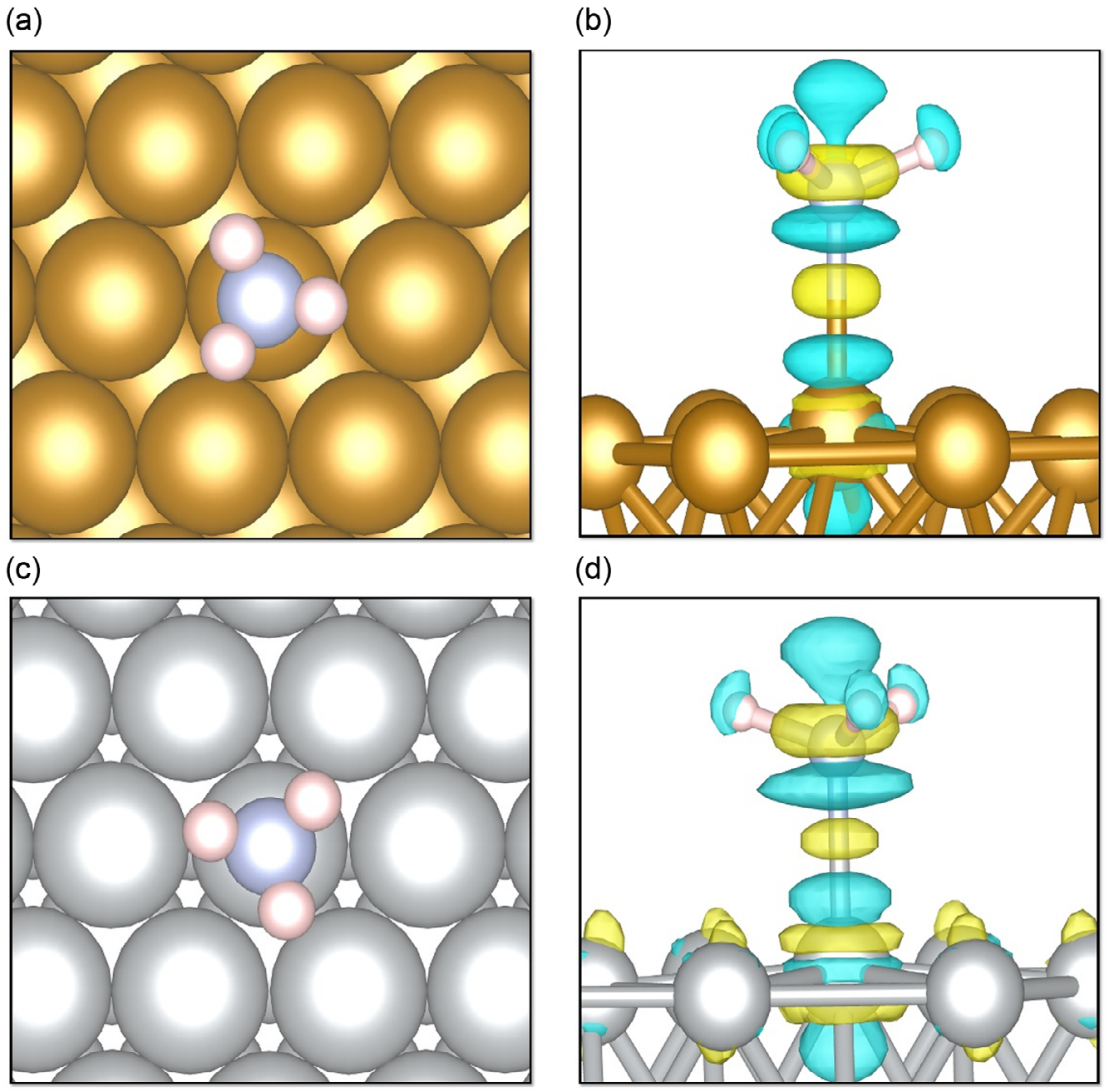
NH_3_ adsorption conformations and the corresponding charge density differences at (a,b) Fe atop site on the Fe(110) and (c,d) Ni atop site on the Ni(111) surfaces. The H, N, Ni, Fe atoms have pink, blue, grey, and brown colours, respectively. Charge accumulation and depletion are represented by yellow and blue colours, respectively. The iso‐surface value was chosen to be 0.004 e/bohr [3].

**TABLE 2:** Structural information including average N–H bond distance, the nearest distance between the adsorbate and the substrate, alongside binding energy and Bader charges of ammonia adsorbate Fe(110) and Ni(111) surfaces.

Adsorption position	*d*_(N–H)_, Å	*d*_(surf–ads)_, Å	*E*_ads_, eV	|Δ*q*|, e
Gas phase	1.027	—	—	—
Fe atop	1.023	2.110	−1.20	−0.053
Ni atop	1.027	2.019	−1.04	−0.081

#### 3.6.2 Electronic Structure Analysis

The charge–transfer behaviour of the two NH_3_ adsorption configurations was qualitatively and quantitatively analysed using charge density difference distribution (CDDD) and Bader charge analysis, respectively. As illustrated in the CDDD maps (Figure 14b,d), blue regions surround the H atoms of the NH_3_ molecule, while yellow regions are concentrated around the N atom. These features suggest that the N atom undergoes charge depletion, whereas electron density accumulates around the H atoms. The Bader charge analysis for the NH_3_ molecule and its constituent atoms across the two adsorption configurations is summarized in Tables 2 and S5. A significant electronic interaction between NH_3_ and the Fe(110) and Ni(111) surfaces is observed, corresponding to Bader charge transfers (Δ*q*) of −0.053 *e* and−0.081 *e*, respectively. Overall, the negative Bader charge of NH_3_ across the two adsorption configurations indicates a net electron transfer from the ammonia molecule to the catalytic surface, indicating that NH_3_ acts as an electron donor. Furthermore, the projected density of states (PDOS) of the ammonia adsorbate and the d‐orbital of Fe(110) and Ni(111) metallic surfaces was calculated for the two adsorption structures (Figure 15a–b). The 1S orbital of H atom and 2p orbital of N atom, of the ammonia molecule, occupy electronic states at a *E*–*E*_f_ value of around −9.0 eV for the two configurations. The least DOS contribution was observed from the H‐1s orbitals in both adsorption configurations, which can be attributed to the dominant role of the N atom in the NH_3_–surface interaction.

**FIGURE 15:**
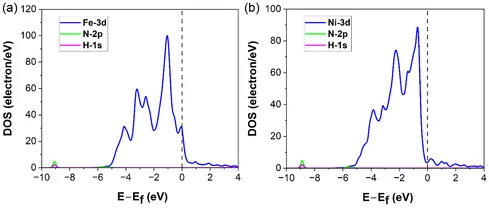
PDOS for the NH_3_ adsorption conformations over the (a) Fe(110) and (b) Ni(111) surfaces. The dashed line indicates the Fermi energy level (*E*_f_), located at 0 eV.

#### 3.6.3 Mechanism of Ammonia Decomposition Reaction

The reaction mechanism of NH_3_ decomposition was examined to explain the enhanced activity of the Fe(110) surface, present in the reduced HEO anodes, in comparison with the Ni(111) surface. The ammonia cracking reaction is represented as follows:

(1) $${\mathrm{NH}}_{3}\;\to \;\frac{3}{2}H_{2}+\frac{1}{2}N_{2}$$

The well‐established reaction mechanism of ammonia cracking involves ammonia adsorption (R1), followed by sequential dehydrogenation of NH_*x*_ (*x* = 0–3) intermediates (R2–R4), and eventually the desorption of nitrogen and hydrogen to the gas phase (R5–R7) [80, 83]. The configurations associated with the initial (IS), transition (TS), and final states (FS) of each reaction step on the Fe(110) and Ni(111) are illustrated in Figures 16a–d and 17a–d, respectively, while the corresponding forward (*E*_a,f_) and backward (*E*_a,b_) activation energies, alongside reaction energy (Δ*H*) are listed in Table 3. The ammonia adsorption follows chemisorption mode (R1) on both surfaces, as confirmed by the strong adsorption energies. Subsequently, the chemisorbed NH_3_ molecule is dissociated into NH_2_* and H* derivatives (R2), which are coordinated to the bridge sites and three‐fold hollow sites, respectively, in the FS structure on both Fe(110) and Ni(111) surfaces. This elementary step overcomes considerably lower kinetic barrier of 0.83 eV on the Fe(110) surface with reference to the Ni(111) counterpart (1.27 eV). This step is also thermodynamically more favourable on the Fe(110) due to its high exothermic reaction energy (−0.50 eV), whereas it is endothermic on the Ni(111) substrate (0.09 eV). Then, the NH_2_* intermediate followed dehydrogenation reaction to form NH* and H* species (R3), which are bonded to hollow sites in the FS configuration on both surfaces. This accounts for an activation energy of 0.43 eV on the Fe(110), which is nearly half of that on the Ni(111) surface (0.90 eV), implying that this step is kinetically more facile on the Fe(110) substrate. This step releases higher reaction energy (−0.96 eV) on the Fe(110) relative to the Ni(111) surface (−0.18 eV). Next, further deprotonation of the NH* intermediate (R4) occurs to form H* and N* species at relatively high activation energies of 1.19 and 1.31 eV on the Fe(110) and Ni(111) surfaces, respectively. This elementary reaction requires a reaction energy of 0.03 and 0.89 eV on the Fe(110) and Ni(111) surfaces, signalling that this step is both kinetically and thermodynamically unfavourable on both surfaces. Afterwards, the desorption of hydrogen adatoms (R5) into the gas phase was found to be barrierless on both surfaces. The formation of the N_2_* intermediate (R6) via recombination of N* atoms is expected to be the rate‐limiting step for NH_3_ decomposition on Fe(110) and Ni(111) given that it has the largest activation barriers of 2.43 and 1.63 eV, respectively, among all elementary steps examined. This is consistent with the results of Zhang et al. [80], who investigated the NH_3_ cracking mechanism and reported that the associative N_2_ desorption step demonstrated the greatest activation energy of 1.80 eV among all steps on FeNi(111), a model representing the Fe‐modified Ni/BZCYYb anode. Moreover, our previous contributions [4, 83] revealed that the association of N* adatoms is the rate‐limiting step for the NH_3_ decomposition on the stoichiometric as well as locally oxygen‐enriched Ni–YSZ anodes. This substantial activation barrier for associative nitrogen desorption might induce partial nitrogen accumulation on the surface, which may cause surface poisoning of the HEO anodes. The minimum energy path (MEP) of NH_3_ decomposition (Figure 18) shows that, except for R6, all stepwise NH_*x*_ dehydrogenation steps (R2–R4) proceed more easily on the Fe(110) surface than on Ni(111).

**FIGURE 16:**
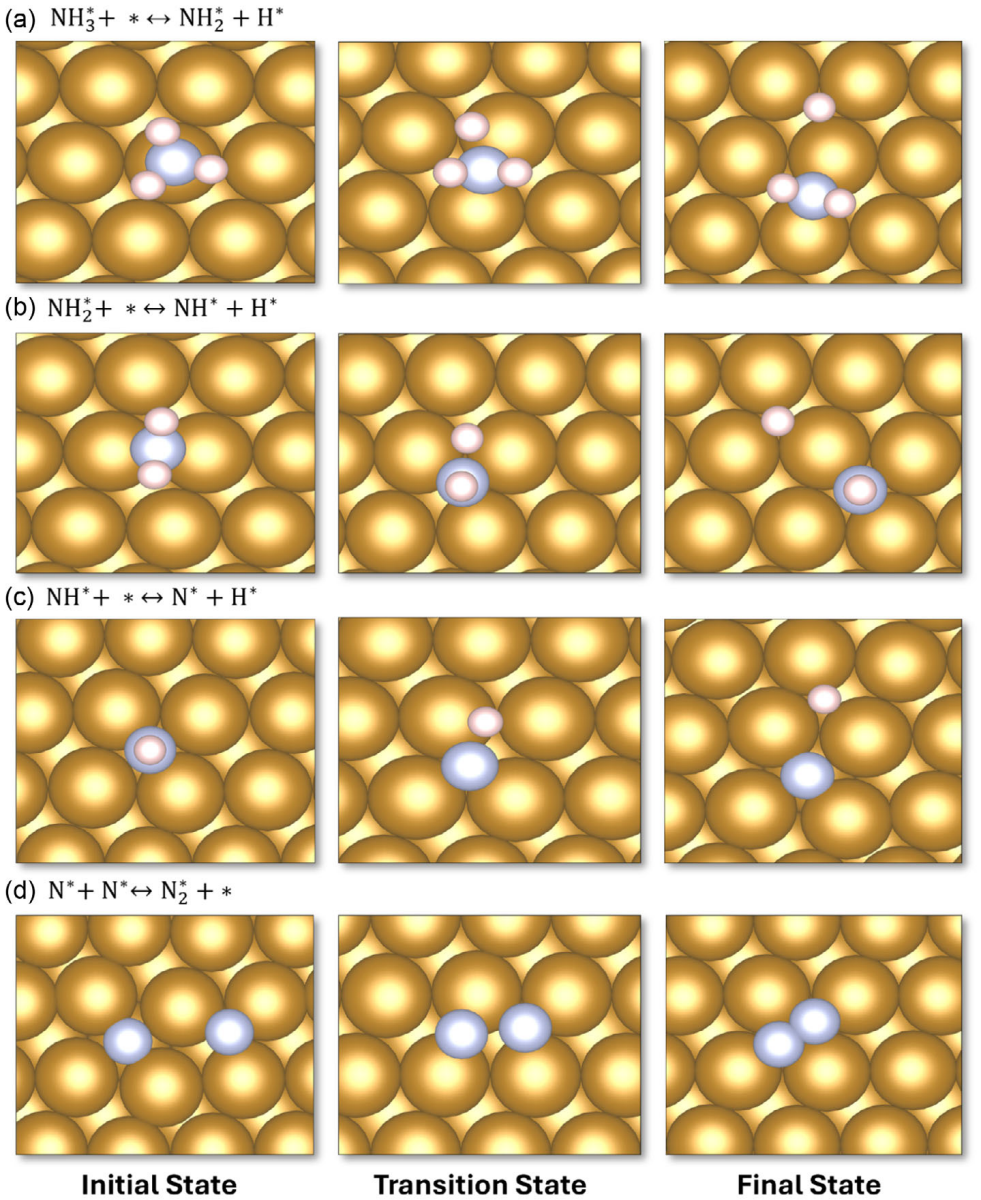
Initial, transition, and final state configurations of elementary steps (R2, R3, R4, and R6) related to NH_3_ decomposition steps on the Fe(110).

**FIGURE 17:**
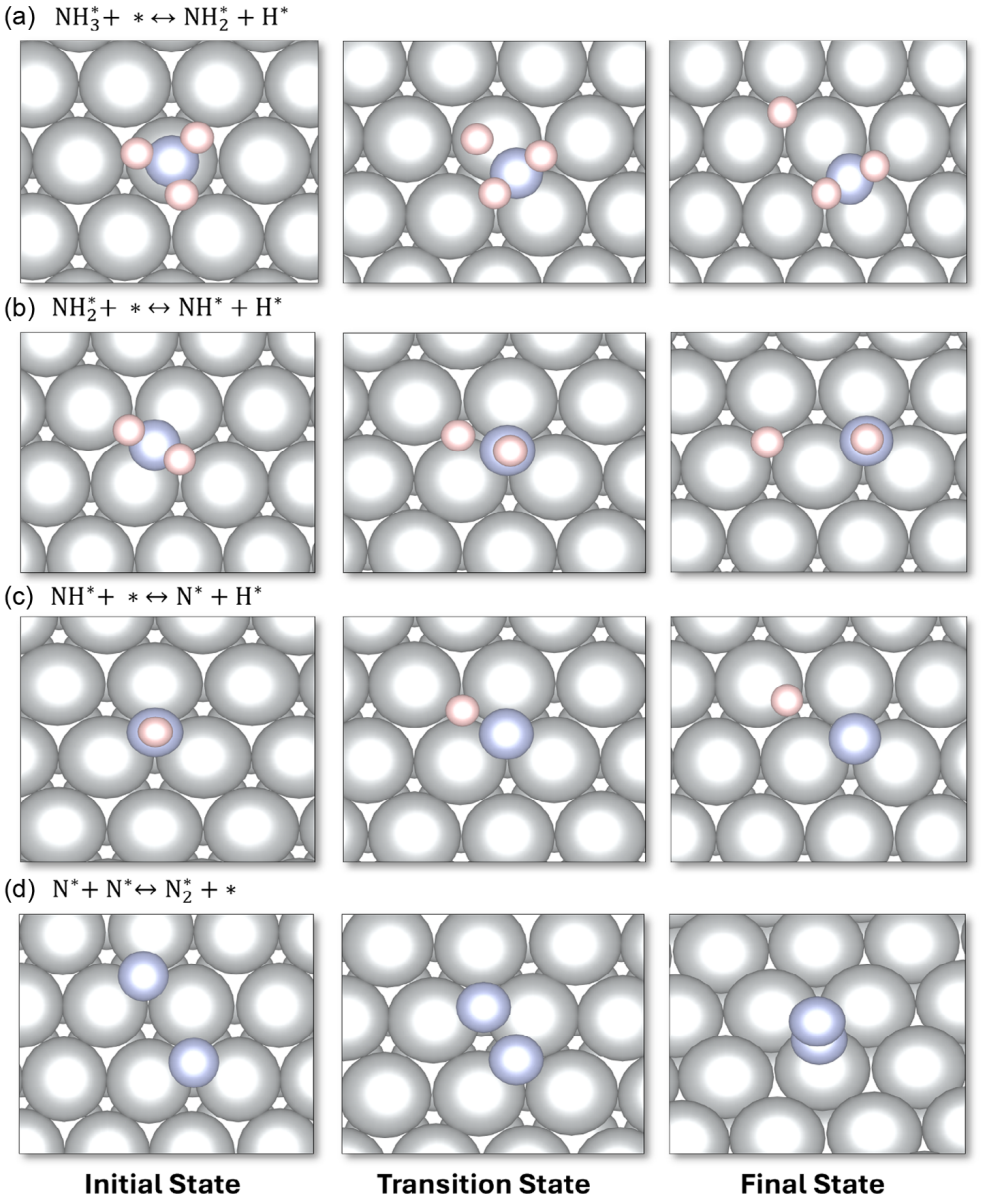
Initial, transition, and final state configurations of elementary steps (R2, R3, R4, and R6) related to NH_3_ decomposition steps on Ni(111).

**TABLE 3:** The forward (*E*_a,f_), and backward (*E*_a,b_) activation energies, along with the reaction energy (Δ*H*), associated with the elementary steps related to NH_3_ cracking on the Fe(110) and Ni(111) slabs.

Step	Reaction	*E*_a,f_, eV		*E*_a,b_, eV		Δ*H*, eV	
		Fe(110)	Ni(111)	Fe(110)	Ni(111)	Fe(110)	Ni(111)
R1	NH_3(g)_ + * ↔ NH_3_*	—	—	—	—	−1.20	−1.04
R2	NH_3_* + * ↔ H* + NH_2_*	0.83	1.27	1.33	1.18	−0.50	0.09
R3	NH_2_* + * ↔ H* + NH*	0.43	0.90	1.40	1.08	−0.96	−0.18
R4	NH* + * ↔ H* + N*	1.19	1.31	1.16	0.42	0.03	0.89
R5	2H* ↔ H_2(g)_ + 2*	0	0	0	0	1.53	1.19
R6	2N* ↔ N_2_* + *	2.43	1.63	1.09	2.18	1.34	−0.55
R7	N_2_* ↔ N_2(g)_ + *	—	—	—	—	1.38	0.61

**FIGURE 18:**
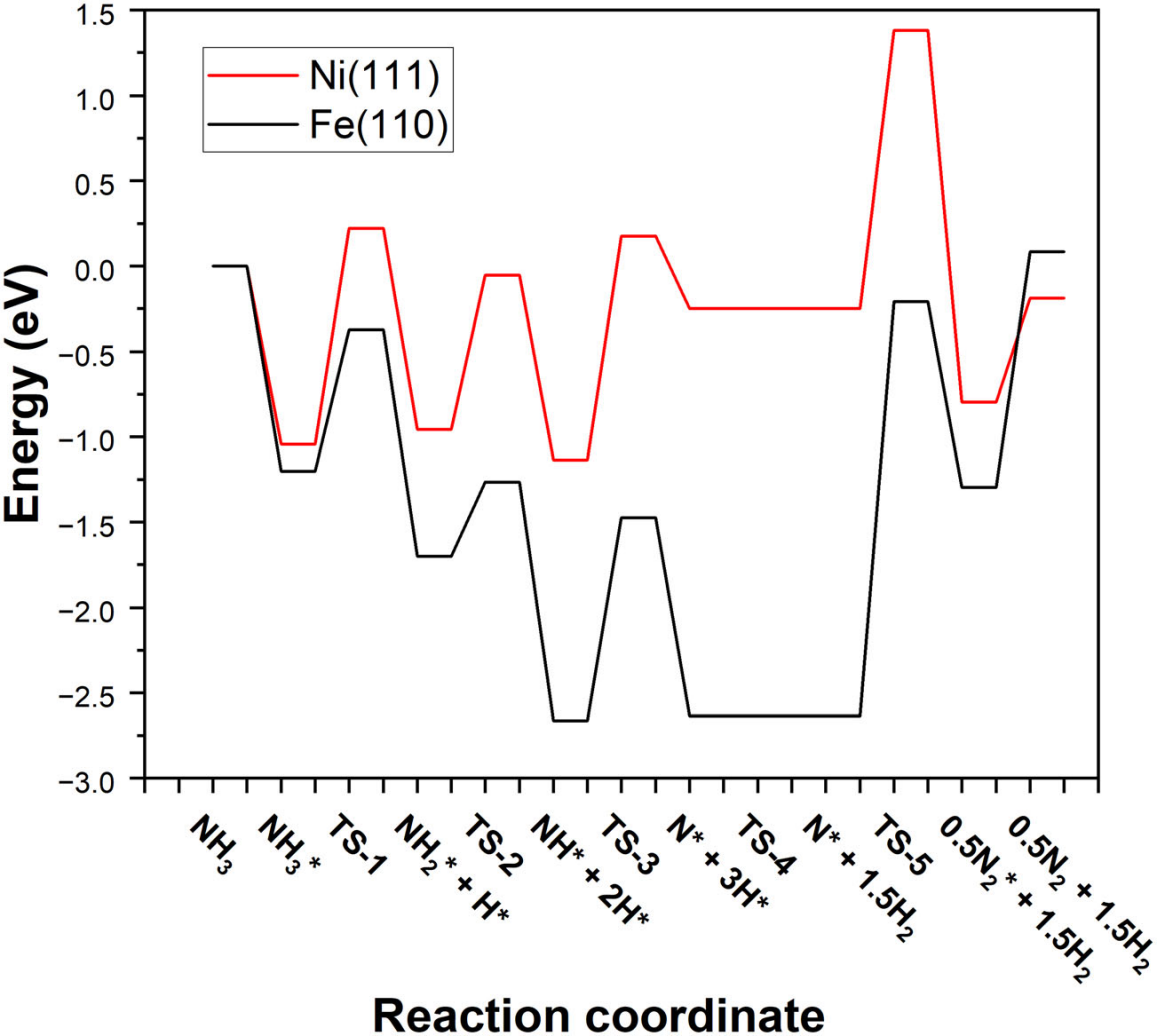
Minimum energy path (MEP) of NH_3_ decomposition reaction on the Fe(110) and Ni(111) slabs. All energies are referenced relative to an NH_3_ molecule in the gas‐phase.

## 4 Conclusion

In this work, high‐entropy spinel ferrite materials, (M_0.3_Ni_0.21_Cu_0.21_Co_0.14_Mn_0.14_)Fe_2_O_4_ (M = Fe, Mg, or Zn), were successfully prepared via a sol–gel technique. The synchrotron‐based XAS analysis revealed that Fe^3+^, Cu^2+^, Ni^2+^, and Co^2+^ cations were present in both octahedral and tetrahedral coordination environments in the fresh d‐HEO(Fe) sample, while Mn^3+^ ions were predominantly located at octahedral sites. Furthermore, the presence of the *A*_1g_ Raman mode between 669 and 677 cm^−1^ confirms that all three fresh HEO samples possess a partially inverse spinel structure. Nevertheless, the XRD results showed that the spinel oxide structure of all HEO samples underwent complete decomposition after reduction in an H_2_ atmosphere at 900 °C for 16 h, resulting predominantly in the formation of a monometallic Fe phase. The cell having d‐HEO(Fe) anode demonstrated superior electrochemical performance of the DA–SOFC, achieving a maximum PPD of 382.0 mW/cm^2^ at 850 °C, outperforming HEO(Zn) (218.3 mW/cm^2^) and HEO(Mg) (195.7 mW/cm^2^) counterparts. The superior electrochemical performance of d‐HEO(Fe) can be ascribed to its lowest ohmic resistance and improved ammonia cracking kinetics, which are facilitated by its higher Fe content, a known active phase for ammonia decomposition. On the contrary, the lower performance of HEO(Zn) and HEO(Mg) anodes is attributed to Fe particle coarsening, as evidenced by larger Fe particle sizes and reduced surface area of the Fe phase from micro‐CT analysis. The OCV analysis as a function of temperature reveals that ammonia utilization in DA–SOFCs with d‐HEO(Fe) and HEO(Mg) anodes proceeds via a combination of direct NH_3_ electro‐oxidation and indirect—where ammonia first decomposes into hydrogen and nitrogen, followed by H_2_ oxidation—reaction pathway. However, the indirect pathway appears to be the dominant reaction route on the HEO(Zn) anode under the studied conditions. The DFT calculations revealed that the formation of the N_2_* intermediate (R6) via recombination of N* atoms is the rate‐limiting step for ammonia cracking on both Fe(110) and Ni(111) surfaces. This study highlights the feasibility of employing non‐equimolar HEOs as cermet anode materials for DA–SOFC applications.

## Funding

The authors have nothing to report.

## Conflicts of Interest

The authors declare no conflicts of interest.

## Supporting information

Characterization methodology, computational models of Fe(110) and Ni(111) slabs, Rietveld XRD refinement for the fresh and reduced HEO samples, Raman peak positions and their corresponding vibrational assignments, ICP‐OES measured elemental composition of the fresh samples, the raw and segmented 2D slices of the reduced anodes, and Bader charge analysis for the various NH_3_ adsorption conformations.

## Data Availability

The data that support the findings of this study are available from the corresponding author upon reasonable request.

## References

[1] A. H. Tariq , S. A. A. Kazmi , M. Hassan , S. A. Muhammed Ali , and M. Anwar , “Analysis of Fuel Cell Integration with Hybrid Microgrid Systems for Clean Energy: A Comparative Review,” International Journal of Hydrogen Energy 52 (2024): 1005–1034, 10.1016/j.ijhydene.2023.07.238.

[2] A. C. Lavernia , Integration of Power and Cooling for Data Centers by Solid Oxide Fuel Cells and Absorption Chillers (UC Irvine, 2022).

[3] F. Kullmann , A. Schwiers , M. Juckel , N. H. Menzler , and A. Weber , “Enhancement of Performance and Sulfur Tolerance of Ceria‐Based Fuel Electrodes in Low Temperature SOFC,” Journal of the Electrochemical Society 171, no. 4 (2024): 044511, 10.1149/1945-7111/ad3ebd.

[4] O. Elmutasim , D. S. Dhawale , S. Giddey , G. Paul , and S. Bhattacharya , “Unravelling the Reaction Mechanism on Ni–YSZ Anode Supported Direct Ammonia Solid Fuel Cell: Experimental and Theoretical Studies,” Applied Surface Science 686 (2025):162175, 10.1016/j.apsusc.2024.162175.

[5] M. P. Pinczes , D. S. Dhawale , O. Elmutasim , S. Giddey , and S. Bhattacharya , “Hydrogenous Solid Oxide Fuel Cells: Role and Evolution of Anode Materials,” Progress in Materials Science 161 (2026): 101690, 10.1016/j.pmatsci.2026.101690.

[6] L. Hamid , O. Elmutasim , D. S. Dhawale , S. Giddey , and G. Paul , “Comparative Electrochemical Performance of Solid Oxide Fuel Cells: Hydrogen vs. Ammonia Fuels—A Mini Review,” Processes 13, no. 4 (2025): 1145, 10.3390/pr13041145.

[7] N. Zheng , Q. Zhang , Y. Ren , et al., “Thermodynamic Analysis of a Novel SOFC‐Based CCHP System Integrated Supercritical Carbon Dioxide and Organic Rankine Cycles for Waste Heat Cascade Utilization,” Energy 328 (2025): 136547, 10.1016/j.energy.2025.136547.

[8] O. Elmutasim , S. Giddey , D. S. Dhawale , and S. Bhattacharya , Ammonia to Power: Advancing Direct Ammonia Solid Oxide Fuel Cells Through Experimental and Theoretical Studies (Elsevier Ltd., 2024), 192–209, 10.1016/j.ijhydene.2024.11.320.

[9] H. Li , W. Wei , T. Zhang , et al., “Degradation Mechanisms and Mitigation Strategies of Direct Methane Solid Oxide Fuel Cells,” Applied Energy 359 (2024): 122609, 10.1016/j.apenergy.2023.122609.

[10] S. Sengodan , R. Lan , J. Humphreys , et al., Advances in Reforming and Partial Oxidation of Hydrocarbons for Hydrogen Production and Fuel Cell Applications (Elsevier Ltd., 2018), 761–780, 10.1016/j.rser.2017.09.071.

[11] S. Dutta , “A Review on Production, Storage of Hydrogen and Its Utilization as an Energy Resource,” Journal of Industrial and Engineering Chemistry 20, no. 4 (2014): 1148–1156, 10.1016/j.jiec.2013.07.037.

[12] S. Giddey , S. P. S. Badwal , C. Munnings , and M. Dolan , “Ammonia as a Renewable Energy Transportation Media,” ACS Sustainable Chemistry & Engineering 5, no. 11 (2017): 10231–10239, 10.1021/acssuschemeng.7b02219.

[13] Y. Kojima and M. Yamaguchi , “Ammonia as a Hydrogen Energy Carrier,” International Journal of Hydrogen Energy 47, no. 54 (2022): 22832–22839, 10.1016/j.ijhydene.2022.05.096.

[14] D. S. Dhawale , S. Biswas , G. Kaur , and S. Giddey , Challenges and Advancement in Direct Ammonia Solid Oxide Fuel Cells: A Review (Royal Society of Chemistry, 2023), 6176–6192, 10.1039/d3qi01557b.

[15] H.‐T. Huang , D.‐C. Lu , C.‐X. Li , and S.‐L. Zhang , “Advanced Design Strategies for Highly Efficient and Durable Anodes in Direct Ammonia Solid Oxide Fuel Cells,” International Journal of Hydrogen Energy 87 (2024): 89–99, 10.1016/j.ijhydene.2024.09.010.

[16] C. M. Rost , E. Sachet , T. Borman , et al., “Entropy‐Stabilized Oxides,” Nature Communications 6, no. 1 (2015): 8485, 10.1038/ncomms9485. PMC4598836 26415623

[17] Y. Zhang , Z. Wang , L. Wang , and L. Zong , “Ultra‐Small High‐Entropy Alloy as Multi‐Functional Catalyst for Ammonia Based Fuel Cells,” Small 20, no. 43 (2024): 2400892, 10.1002/smll.202400892. 38953333

[18] Y. Luo , J. Li , X. Zhou , et al., “Metal‐Organic Framework‐Derived Non‐Equimolar Ratio Inverse Spinel‐Structured High‐Entropy Oxides as Anode Materials for High‐Performance Lithium‐Ion Batteries,” Journal of Alloys and Compounds 1008 (2024): 176515, 10.1016/j.jallcom.2024.176515.

[19] Z. Chen , B. Ma , C. Dang , J. Che , L. Cheng , and Y. Zhou , “A Novel Strategy of Entropy Engineering at the a‐Site in Spinel Oxides for Developing High‐Performance SOFC Cathodes,” Journal of Materials Chemistry A 12, no. 37 (2024): 24997–25010, 10.1039/D4TA04925J.

[20] V. Prabhahari , R. Praveena , and K. S. Babu , “Novel Spinel Based High Entropy Oxide as Electrode for Symmetric SOFCs,” Journal of Alloys and Compounds 986 (2024): 174152, 10.1016/j.jallcom.2024.174152.

[21] Z. Lin , B. Ma , Z. Chen , and Y. Zhou , “Nanostructured Spinel High‐Entropy Oxide (Fe_0.2_Mn_0.2_Co_0.2_Ni_0.2_Zn_0.2_)_3_O_4_ as a Potential Cathode for Solid Oxide Fuel Cells,” Ceramics International 49, no. 14 (2023): 23057–23067, 10.1016/j.ceramint.2023.04.131.

[22] Y. Liao , F. Zhu , X. Zhang , et al., “An Active High‐Entropy Air Electrode for Enhanced Reversible Solid Oxide Cell Performance and Stability,“ Materials Reports: Energy 5 (2025): 100381, 10.1016/j.matre.2025.100381.

[23] Y. Liu , R. Lin , B. Guo , et al., “Ultrafast Joule Heating Technology for Functional Nanomaterials Synthesis: Recent Progress, Challenges, and Perspectives,” Materials Reports: Energy 5, no. 4 (2025): 100377, 10.1016/j.matre.2025.100377 doi:.

[24] Y. Wang , H. Li , H. Liu , L. Yang , and C. Zeng , “Preparation and Formation Mechanism of Cr‐Free Spinel‐Structured High Entropy Oxide (MnFeCoNiCu)_3_O_4_ ,” Ceramics International 49, no. 2 (2023): 1940–1946, 10.1016/j.ceramint.2022.09.159.

[25] Y. Yu , S. Liu , Y. Ren , et al., “Composition‐Constrained Rocksalt Structure High Entropy Oxides With Non‐Equimolar Cations: Design and Synthesis Based on Cluster‐Plus‐Glue‐Atom Model,” Ceramics International 48, no. 17 (2022): 25647–25650, 10.1016/j.ceramint.2022.05.265.

[26] Y. Liu , J. Hou , C. Cheng , et al., “The Effect of Non‐Equimolar Doping on the Preparation and Electrical Conductivity of Sr(Ti, Zr, Zn, Sn, Hf)O_3_‐σ High Entropy Perovskite Oxide,” Ceramics International 49, no. 13 (2023): 21546–21554, 10.1016/j.ceramint.2023.03.290.

[27] G. Ma , D. Chen , S. Ji , et al., “Medium‐Entropy SrV_1_ _/3_Fe_1_ _/3_Mo_1_ _/3_O_3_ With High Conductivity and Strong Stability as SOFCs High‐Performance Anode,” Materials 15, no. 6 (2022): 2298, 10.3390/ma15062298. PMC8953221 35329750

[28] O. Rahumi , Y. Yuferov , L. Meshi , N. Maman , and K. Borodianskiy , “Ni‐Doping Strategy for Perovskite Anodes Towards High‐Performance Ammonia‐Fueled SOFCs,” Journal of Power Sources 631 (2025): 236320, 10.1016/j.jpowsour.2025.236320.

[29] S. Y. Batool , M. H. Hassan , S. U. Rehman , et al., “Leveraging Ni Ex‐Solved Perovskite Structure to Design a Ternary‐Phase‐Catalyzed Composite Anode for High‐Performance Direct Ammonia Solid Oxide Fuel Cells,” ACS Applied Energy Materials 8, no. 4 (2025): 2452–2464, 10.1021/acsaem.4c03038.

[30] S. Zhu , J. Fan , Z. Li , et al., “Metal Exsolution From Perovskite‐Based Anodes in Solid Oxide Fuel Cells,” Chemical Communications 60 (2024): 1062–1071, 10.1039/d3cc05688k. 38167745

[31] Y. H. Kim , H. Jeong , B. R. Won , et al., “Nanoparticle Exsolution on Perovskite Oxides: Insights into Mechanism, Characteristics and Novel Strategies,” Nano‐Micro Letters 16 (2024): 33, 10.1007/s40820-023-01258-4. PMC10684483 38015283

[32] Y. Yoon , Y. Choi , Y. Bae , J. Hong , and K. Kim , “Ni‐Based Alloy Catalysts for Cost‐Effective Hydrogen Production From Ammonia Decomposition,” Applied Surface Science Advances 27 (2025): 100759, 10.1016/j.apsadv.2025.100759.

[33] F. Zurlo , A. Iannaci , V. M. Sglavo , and E. Di Bartolomeo , “Copper‐Based Electrodes for IT‐SOFC,” Journal of the European Ceramic Society 39, no. 1 (2019): 17–20, 10.1016/j.jeurceramsoc.2018.02.029.

[34] J. Geng , Q. Guo , J. Pan , B. Chi , and J. Pu , “Improvement of Fe Addition on Redox Stability of Ni/YSZ Composite Anode for Intermediate Temperature Solid Oxide Fuel Cell,” Renewable Energy 240 (2025): 122236, 10.1016/j.renene.2024.122236.

[35] O. Elmutasim , D. S. Dhawale , M. Pinczes , et al., “Deciphering Ni Content–Induced Microstructural Evolution in Ni–YSZ Cermets for Direct Ammonia SOFC Performance,” ACS Applied Materials & Interfaces 17, no. 47 (2025): 65225–65241, 10.1021/acsami.5c12421. PMC12673522 41220380

[36] B. Ravel , and M. Newville , “ *ATHENA*, *ARTEMIS*, *HEPHAESTUS* : Data Analysis for X‐Ray Absorption Spectroscopy Using *IFEFFIT* ,” Journal of Synchrotron Radiation 12, no. 4 (2005): 537–541, 10.1107/S0909049505012719. 15968136

[37] J. P. Perdew , K. Burke , and M. Ernzerhof , “Generalized Gradient Approximation Made Simple,” Physical Review Letters 77, no. 18 (1996): 3865–3868, 10.1103/PhysRevLett.77.3865. 10062328

[38] P. E. Blöchl , “Projector Augmented‐Wave Method,” Physical Review B 50, no. 24 (1994): 17953–17979, 10.1103/PhysRevB.50.17953. 9976227

[39] O. Elmutasim and S. M. Alhassan , “Unraveling the Role of Surface Termination in Ni2P(001) for the Direct Desulfurization Reaction of Dibenzothiophene (DBT): A Density Functional Theory (DFT) and Microkinetic Study,” Industrial & Engineering Chemistry Research 60, no. 43 (2021): 15525–15539, 10.1021/acs.iecr.1c03219.

[40] O. Elmutasim , L. M. Maghrabi , D. S. Dhawale , and K. Polychronopoulou , “Engaging the Concepts of Bimetallicity and Mechanical Strain for N_2_ Activation: A Computational Exploration,” ACS Applied Materials & Interfaces 16, no. 41 (2024): 56254–56270, 10.1021/acsami.4c09691. PMC11492178 39370605

[41] O. Elmutasim and K. Polychronopoulou , “Direct Deoxygenation Reaction of Oxygenated Model Compounds by Biomass Pyrolysis on the Ni5P4(001) Surface: A Computational Study,” Sustainable Energy & Fuels 7, no. 6 (2023): 1415–1432, 10.1039/D2SE01521H.

[42] G. Tanimu , O. Elmutasim , H. Alasiri , and K. Polychronopoulou , “Unravelling the Pathway for the Dehydrogenation of N‐Butane to 1,3‐Butadiene Using Thermodynamics and DFT Studies,” Chemical Engineering Science 280 (2023): 119059, 10.1016/j.ces.2023.119059.

[43] L. Al Ali , S. Alkhoori , O. Elmutasim , K. Al‐Ali , and K. Polychronopoulou , “Computational Insights Into the Deoxygenation Reaction of Biomass‐Derived Phenolic Compounds Over Ni2P (001) Catalysts,” Materials Chemistry and Physics 328 (2024): 129926, 10.1016/j.matchemphys.2024.129926.

[44] S. He , J. Lucio‐Vega , L. Zhang , et al., “Integrating Visualization Methods With Chemical Kinetics Model Solution and Editing Tools,” Energy & Fuels 31, no. 9 (2017): 9881–9889, 10.1021/acs.energyfuels.7b01202.

[45] O. Elmutasim , G. Basina , D. A. Shami , et al., “On the Impact of Copper Local Environment on Hydrogen Sulfide Adsorption Within Microporous AlPO4‐5,” Journal of Environmental Chemical Engineering 8, no. 5 (2020): 104245, 10.1016/j.jece.2020.104245 doi:.

[46] O. Elmutasim , G. Tanimu , I. H. Aljundi , and S. Al‐Khattaf , “Bimetallic Bi‐Ni Oxides Over Carbide Supports for Oxidative Dehydrogenation of n‐Butane: Experimental and Kinetic Modelling,” The Canadian Journal of Chemical Engineering 96, no. 6 (2018): 1367–1376, 10.1002/cjce.23123.

[47] J. Ko , H. Kwon , H. Kang , B.‐K. Kim , and J. W. Han , “Universality in Surface Mixing Rule of Adsorption Strength for Small Adsorbates on Binary Transition Metal Alloys,” Physical Chemistry Chemical Physics 17, no. 5 (2015): 3123–3130, 10.1039/C4CP04770B. 25515855

[48] Z. Xing , Z. Ju , J. Yang , H. Xu , and Y. Qian , “One‐Step Solid State Reaction to Selectively Fabricate Cubic and Tetragonal CuFe_2_O_4_ Anode Material for High Power Lithium Ion Batteries,” Electrochimica Acta 102 (2013): 51–57, 10.1016/j.electacta.2013.03.174.

[49] T. U. Tran Thi , V. H. Phan , H. T. Pham Nguyen , T. L. Nguyen , A. N. Vu , and T. K. Le , “Synthesis of Magnetic CuFe_2_O_4_/Fe_2_O_3_ Core‐Shell Materials and Their Application in Photo‐Fenton‐Like Process With Oxalic Acid as a Radical‐Producing Source,” Journal of Asian Ceramic Societies 9, no. 3 (2021): 1091–1102, 10.1080/21870764.2021.1939241.

[50] L. Xu , L. Wang , Y. Li , et al., “A Novel High‐Entropy Spinel Ferrites (CoNiCuZnMg)Fe_2_O_4_ Catalyst for H_2_ Production via Steam Reforming of Derived Volatiles From Polypropylene and Waste Cooking Oil,” Chemical Engineering Journal 488 (2024): 150767, 10.1016/j.cej.2024.150767 doi:.

[51] P. Chand , S. Vaish , and P. Kumar , “Structural, Optical and Dielectric Properties of Transition Metal (MFe_2_O_4_; M = Co, Ni and Zn) nanoferrites,” Physica B: Condensed Matter 524 (2017): 53–63, 10.1016/j.physb.2017.08.060.

[52] P. R. Kumar , Y. H. Jung , K. K. Bharathi , C. H. Lim , and D. K. Kim , “High Capacity and Low Cost Spinel Fe_3_O_4_ for the Na‐Ion Battery Negative Electrode Materials,” Electrochimica Acta 146 (2014): 503–510, 10.1016/j.electacta.2014.09.081.

[53] K. B. Modi , P. Y. Raval , S. J. Shah , et al., “Raman and Mossbauer Spectroscopy and X‐Ray Diffractometry Studies on Quenched Copper–Ferri–Aluminates,” Inorganic Chemistry 54, no. 4 (2015): 1543–1555, 10.1021/ic502497a. 25594232

[54] N. Sharma , S. Mandal , and S. Marik , “Raman Spectroscopy, and Magnetic Properties of New Al, Ga, and Mn‐Based High Entropy Oxides,” Ceramics International 50, no. 22, Part C (2024): 48200–48209, 10.1016/j.ceramint.2024.09.170.

[55] Z. Luo and X.‐P. Zhou , “Liquid Metal–Induced Low‐Temperature Synthesis of Tunable High‐Entropy Oxides,” Science Advances 11, no. 23 (2025): eadw1461, 10.1126/sciadv.adw1461. PMC12143384 40479076

[56] A. Wang , K. E. Kuebler , B. L. Jolliff , and L. A. Haskin , “Raman Spectroscopy of Fe‐Ti‐Cr‐Oxides, Case Study: Martian Meteorite EETA79001,” American Mineralogist 89, no. 5‐6 (2004): 665–680, 10.2138/am-2004-5-601.

[57] Q. Qin , Y. Liu , X. Li , T. Sun , and Y. Xu , “Enhanced Heterogeneous Fenton‐Like Degradation of Methylene Blue by Reduced CuFe_2_O_4_ ,” RSC Advances 8, no. 2 (2018): 1071–1077, 10.1039/C7RA12488K. PMC9076977 35538971

[58] A. M. Hengne , A. K. Samal , L. R. Enakonda , et al., “Ni–Sn‐Supported ZrO_2_ Catalysts Modified by Indium for Selective CO_2_ Hydrogenation to Methanol,” ACS Omega 3, no. 4 (2018): 3688–3701, 10.1021/acsomega.8b00211. PMC6641425 31458617

[59] S. Maiti , A. Pramanik , T. Dhawa , M. Sreemany , and S. Mahanty , “Bi‐Metal Organic Framework Derived Nickel Manganese Oxide Spinel for Lithium‐Ion Battery Anode,” Materials Science and Engineering: B 229 (2018): 27–36, 10.1016/j.mseb.2017.12.018.

[60] D. Guo , Z. Wu , Y. An , et al., “Room Temperature Ferromagnetism in (Ga_1−x_Mn_x_)_2_O_3_ Epitaxial Thin Films,” Journal of Materials Chemistry C 3, no. 8 (2015): 1830–1834, 10.1039/C4TC02833C.

[61] A. Ramírez , P. Hillebrand , D. Stellmach , M. M. May , P. Bogdanoff , and S. Fiechter , “Evaluation of MnO_x_, Mn_2_O_3_, and Mn_3_O_4_ Electrodeposited Films for the Oxygen Evolution Reaction of Water,” The Journal of Physical Chemistry C 118, no. 26 (2014): 14073–14081, 10.1021/jp500939d.

[62] M. A. Brisk and A. D. Baker , “Shake‐up Satellites in X‐Ray Photoelectron Spectroscopy,” Journal of Electron Spectroscopy and Related Phenomena 7, no. 3 (1975): 197–213, 10.1016/0368-2048(75)80061-2.

[63] M. Oku , K. Hirokawa , and S. Ikeda , “X‐Ray Photoelectron Spectroscopy of Manganese—Oxygen Systems,” Journal of Electron Spectroscopy and Related Phenomena 7, no. 5 (1975): 465–473, 10.1016/0368-2048(75)85010-9.

[64] V. Krishnan , R. K. Selvan , C. O. Augustin , A. Gedanken , and H. Bertagnolli , “EXAFS and XANES Investigations of CuFe_2_O_4_ Nanoparticles and CuFe_2_O_4_−MO_2_ (M = Sn, Ce) Nanocomposites,” The Journal of Physical Chemistry C 111, no. 45 (2007): 16724–16733, 10.1021/jp073746t.

[65] E. Chalmin , F. Farges , and G. E. Brown , “A Pre‐Edge Analysis of Mn K‐Edge XANES Spectra to Help Determine the Speciation of Manganese in Minerals and Glasses,” Contributions to Mineralogy and Petrology 157, no. 1 (2009): 111–126, 10.1007/s00410-008-0323-z.

[66] T. Zahoransky , A. V. Wegorzewski , W. Huong , and C. Mikutta , “X‐Ray Absorption Spectroscopy Study of Mn Reference Compounds for Mn Speciation in Terrestrial Surface Environments,” American Mineralogist: Journal of Earth and Planetary Materials 108, no. 5 (2023): 847–864, 10.2138/am-2022-8236.

[67] S. M. Masina , G. C. Nkala , M. A. Kiefer , et al., “A Multi‐Pronged Approach to the Determination of Cation Distribution, Site Selectivity, and Chemical Ordering in “High Entropy” Spinel Oxides,” Journal of the American Chemical Society 147, no. 42 (2025): 38283–38290, 10.1021/jacs.5c10967. 41065175

[68] S. Senkale , M. Kamp , S. Mangold , et al., “Multi‐Method Characterization of the High‐Entropy Spinel Oxide Mn_0.2_Co_0.2_Ni_0.2_Cu_0.2_Zn_0.2_Fe_2_O_4_: Entropy Evidence, Microstructure, and Magnetic Properties,” Chemistry–Methods 3, no. 2 (2023): e202200043, 10.1002/cmtd.202200043.

[69] K. Iwase and I. Honma , “High‐Entropy Spinel Oxide Nanoparticles Synthesized via Supercritical Hydrothermal Processing as Oxygen Evolution Electrocatalysts,” ACS Applied Energy Materials 5, no. 8 (2022): 9292–9296, 10.1021/acsaem.2c01751.

[70] F. Caddeo , D. Loche , M. F. Casula , and A. Corrias , “Evidence of a Cubic Iron Sub‐Lattice in t‐CuFe_2_O_4_ Demonstrated by X‐Ray Absorption Fine Structure,” Scientific Reports 8, no. 1 (2018): 797, 10.1038/s41598-017-19045-8. PMC5768695 29335500

[71] T. Ishihara and H. Zhong , “Effects of Fe Addition on the Surface Reaction of the Anode of Intermediate Temperature Solid Oxide Fuel Cells,” Scripta Materialia 65, no. 2 (2011): 108–111, 10.1016/j.scriptamat.2010.08.022.

[72] V. Sonn , A. Leonide , and E. Ivers‐Tiffée , “Combined Deconvolution and CNLS Fitting Approach Applied on the Impedance Response of Technical Ni/8YSZ Cermet Electrodes,” Journal of the Electrochemical Society 155, no. 7 (2008): B675, 10.1149/1.2908860.

[73] G. Cinti , A. Baldinelli , G. Bidini , and L. Barelli , “Electrochemical Impedance Spectroscopy Study on Ammonia‐Fed Solid Oxide Fuel Cells,” Journal of Physics: Conference Series 2385, no. 1 (2022): 012052, 10.1088/1742-6596/2385/1/012052.

[74] K. Miyazaki , H. Muroyama , T. Matsui , and K. Eguchi , “Impact of the Ammonia Decomposition Reaction Over an Anode on Direct Ammonia‐Fueled Protonic Ceramic Fuel Cells,” Sustainable Energy & Fuels 4, no. 10 (2020): 5238–5246, 10.1039/D0SE00841A.

[75] A. Leonide , V. Sonn , A. Weber , and E. Ivers‐Tiffée , “Evaluation and Modeling of the Cell Resistance in Anode‐Supported Solid Oxide Fuel Cells,” Journal of the Electrochemical Society 155, no. 1 (2008): B36, 10.1149/1.2801372.

[76] S. P. Jiang and S. P. S. Badwal , “An Electrode Kinetics Study of H_2_ Oxidation on Ni/Y_2_O_3_–ZrO_2_ Cermet Electrode of the Solid Oxide Fuel Cell,” Solid State Ionics 123, no. 1‐4 (1999): 209–224, 10.1016/S0167-2738(99)00124-1.

[77] P. J. Gasper , Y. Lu , A. Y. Nikiforov , S. N. Basu , S. Gopalan , and U. B. Pal , “Detailed Electrochemical Performance and Microstructural Characterization of Nickel – Yttria Stabilized Zirconia Cermet Anodes Infiltrated With Nickel, Gadolinium Doped Ceria, and Nickel – Gadolinium Doped Ceria Nanoparticles,” Journal of Power Sources 447 (2020): 227357, 10.1016/j.jpowsour.2019.227357.

[78] L. Sun , C. H. Hendon , M. A. Minier , A. Walsh , and M. Dincă , “Million‐Fold Electrical Conductivity Enhancement in Fe_2_(DEBDC) Versus Mn_2_(DEBDC) (E = S, O),” Journal of the American Chemical Society 137, no. 19 (2015): 6164–6167, 10.1021/jacs.5b02897. PMC4442594 25932955

[79] Y. Ya , Y. Xu , Y. Liu , B. Sun , J. Liu , and X. Cheng , “Effect of Ammonia Catalytic Decomposition Coating on Electrochemical Performance in Direct Ammonia Solid Oxide Fuel Cells,” Energy & Fuels 39, no. 4 (2025): 2216–2229, 10.1021/acs.energyfuels.4c04898.

[80] H. Zhang , Y. Zhou , K. Pei , et al., “An Efficient and Durable Anode for Ammonia Protonic Ceramic Fuel Cells,” Energy & Environmental Science 15, no. 1 (2022): 287–295, 10.1039/D1EE02158C.

[81] L. Liu , K. Sun , X. Wu , et al., “Improved Performance of Ammonia‐Fueled Solid Oxide Fuel Cell With SSZ Thin Film Electrolyte and Ni‐SSZ Anode Functional Layer,” International Journal of Hydrogen Energy 37, no. 14 (2012): 10857–10865, 10.1016/j.ijhydene.2012.04.101.

[82] D. R. Lide , CRC Handbook of Chemistry and Physics (CRC Press, 2004).

[83] O. Elmutasim , D. S. Dhawale , M. Pinczes , S. Giddey , G. Paul , and S. Bhattacharya , “Mechanistic Investigation of Ammonia Utilization Pathways on a Locally Oxygen Enriched Ni–YSZ Anode for Direct Ammonia‐Fed Solid Oxide Fuel Cells: An Ab Initio Study,” ACS Sustainable Chemistry & Engineering 13, no. 42 (2025): 18242–18254, 10.1021/acssuschemeng.5c07973.

