# Supplementary material for: Non‐Equimolar High‐Entropy Spinel Oxides as Anode Materials for a Direct Ammonia‐Fed Solid Oxide Fuel Cell
Source: Small Sci. 2026 Oct 21;6(10):e70427. doi: 10.1002/smsc.70427 (PMC13687658; doi:10.1002/smsc.70427)
Supplement: Supplementary file 1 [file SMSC-6-e70427-s001.pdf]

Non-equimolar High-Entropy Spinel Oxides as Anode Materials for a Direct Ammonia-Fed Solid Oxide Fuel Cell

Omer Elmutasim[1,2], Maxwell P. Pinczes[1,2], Dattatray S. Dhawale[1*], Sarbjit Giddey[1], Sankar Bhattacharya[2]
[1] CSIRO Energy, Private Bag 10, Clayton South 3169, Victoria, Australia.
[2] Department of Chemical and Biological Engineering, Monash University, Clayton 3800, Victoria, Australia
Email address: dattatray.dhawale@csiro.au

Characterization methods:

X-ray Diffraction (XRD) was conducted on the HEO (Zn), HEO (Mg) and HEO (Fe) fresh and reduced samples, using a Rigaku SmartLab, with a rotating Cu K_α source (200 mA, 45 kV), scanning over a 2θ range of 5 - 130° at a scan rate of 0.5°/min and a step-size of 0.01°. The microstructure and surface features of these samples was also analysed using Field-emission scanning electron microscopy (FE-SEM) with energy-dispersive X-ray spectroscopy (EDX). The Raman spectroscopy was carried out using Renishaw inVia™ Raman spectrometer having a 514 nm laser source in the wavenumber window of 175–800 cm^{-1}.

These samples were further analysed through Scanning Transmission Electron Microscopy with Energy Dispersive X-ray (STEM-EDX). Samples were prepared for analysis by ultrasonically dispersing the reduced HEO (Zn), HEO (Mg) and HEO (Fe) powders in ethanol, followed by drop-casting the suspension onto holey carbon film–coated copper grids. The High-angle annular dark-field scanning transmission electron microscopy (HAADF-STEM) imaging and energy-dispersive X-ray (EDX) elemental mapping utilised an FEI Tecnai F20 microscope operated at 200 kV equipped with a windowless Bruker X-Flash EDX detector and Esprit software, whereas conventional TEM images were acquired using a Gatan OneView CMOS camera.

Inductively coupled plasma optical emission spectroscopy (ICP-OES) was used to analyse the composition of the HEO through digesting samples with 6mL of 37% HCl and 2mL of 69% HNO3 in plastic hot block tubes. The samples were heated for several hours at 90°C and once the reactions had ceased, were diluted as required and analysed by ICP-OES. Certified multi-element standards were used to check the accuracy of the calibrations and verify the method used.

X-ray Photoelectron Spectroscopy (XPS): XPS measurements of the reduced HEO (Zn), HEO (Mg) and HEO (Fe) samples were conducted via a Thermo Scientific Nexsa Surface Analysis System equipped with a hemispherical analyzer. Monochromatic Al Kα radiation (hν = 1486.6 eV) was operated at a power of 72 W (6 mA, 12 kV), with an X-ray spot size of 400 × 250 μm^2. High-resolution (narrow-scan) spectra were acquired with analyzer pass energies of 50 eV, with step size of 0.1 eV, respectively. The base pressure in the analysis chamber was kept below 5.0×10^{-9} mbar. Surface charging effects were compensated for by using a low-energy dual-beam (ion and electron) flood gun. Spectral analysis and data processing were performed using Avantage software (v6.7.0).

Micro-Computed Tomography (Micro-CT): The three-dimensional microstructure of reduced HEO anodes was explored via lab–based Micro-CT technique. Each anode was first reduced at 900 °C for 24 h under forming gas (5% H_2/N_2) prior to imaging. Micro-CT measurements were carried out using a Zeiss Xradia 515 system equipped with a tungsten-on-diamond window X-ray source. An LE4 filter was used for the HEO(Zn) and HEO(Mg) samples, while an HE4 filter was employed for the d-HEO(Fe) sample. Micro-CT scans of the d-HEO(Fe) sample were performed at a source voltage of 80 kV, source power of 7 W, and an exposure time of 6 s per projection. In contrast, the HEO(Zn) and HEO(Mg) samples were scanned at 110 kV, 10 W, and 20 s per projection. A total of 2401 projections were acquired for each scan, yielding voxel sizes of 0.86 μm for the d-HEO(Fe) sample and 0.80 μm for the HEO(Zn) and HEO(Mg) samples when using a 20× objective lens. Several microstructural features were quantified, including: (i) volume fractions of each anode phase (V_{phase}), (ii) volume-specific surface area (SSA), (iii) volume-specific interfacial contact areas (SIA) , (iv) Triple Phase Boundary density (ρ_{TPB}), and (v) Feret diameters of metallic particles. The software used for data analysis was Avizo Fire (Avizo, Thermo Fisher Scientific, Waltham, Massachusetts, U.S.) along an in-house python code. The calculation methods and post-processing procedures are detailed as below:

Three-Phase Segmentation: The acquired micro-CT datasets were processed in Avizo software, with noise reduction performed using a non-local means filter prior to segmentation. Watershed segmentation was employed to classify the sample into Fe, FeNiCu, MO (M = Mn or Fe), GDC, and pore phases. The resulting segmented microstructure was rendered in 3D using the Volume Rendering module in Avizo.

Spatial Orientation: The orientation of the anode in the three-dimensional volume was described using x, y, and z axes, with the z-axis perpendicular to the anode/electrolyte interface and the x- and y-axes orthogonal to each other and parallel to the interface.

Composition Analysis: Phase volume fractions were determined using Avizo and an in-house Python routine. Given the agreement between the two methods, only one representative dataset is presented.

Particle size: The Feret diameters of all phases were determined using ImageJ.

Triple-Phase Boundary: The total triple-phase boundary density (ρ_{TPB}) along the z-axis were calculated via an in-house Python routine.

Interfacial Contact Area and Surface Area: Phase interfacial contact areas (SIA) and the corresponding volume-specific surface areas (SSA) were quantified using an in-house Python script. The local Fe–GDC interfacial contact area along the z-direction was also determined with the same codes

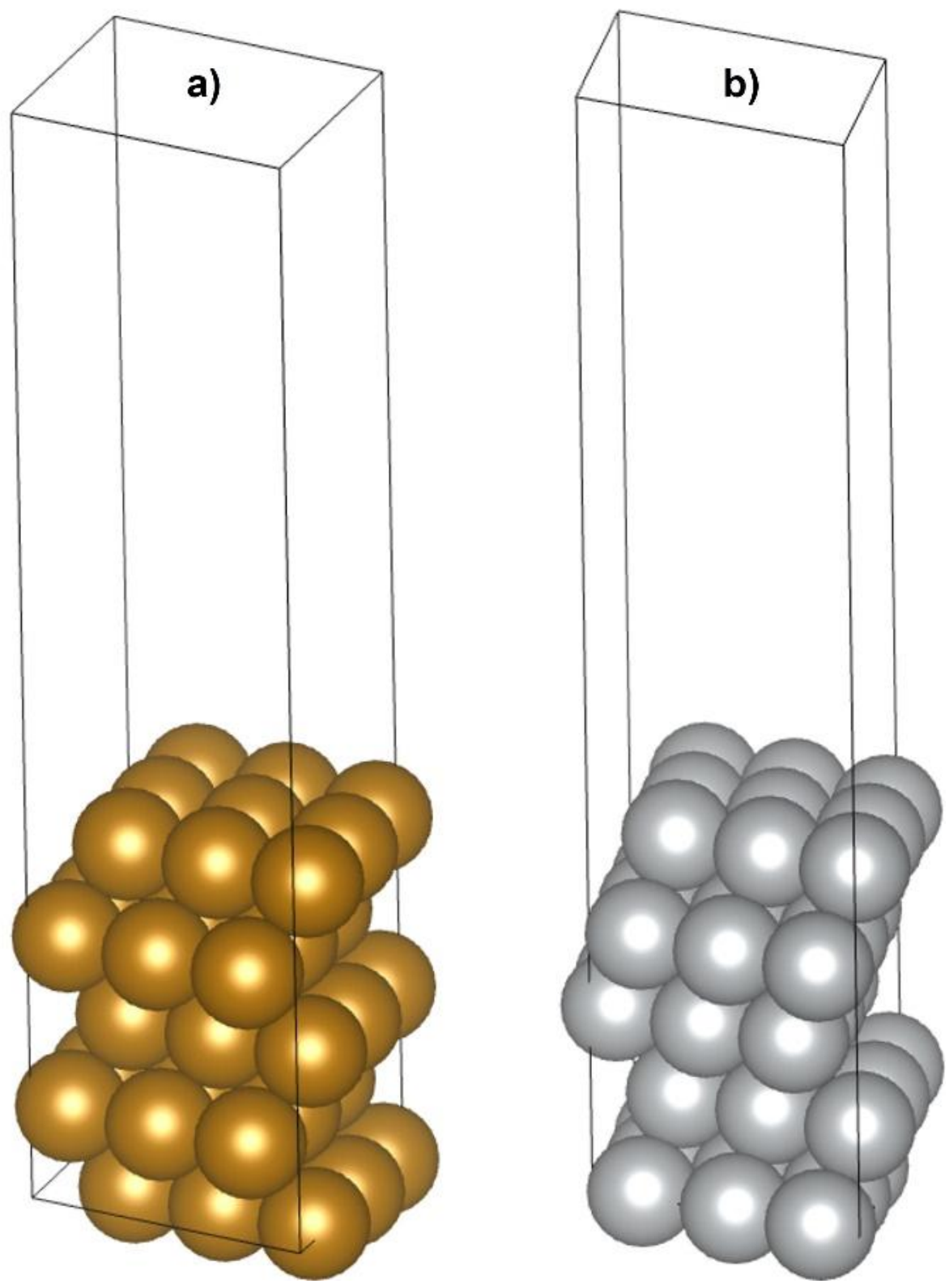

Figure S1: *The (a) Fe(110) and (b) Ni(111) slabs. The nickel, and iron atoms are shown in grey and brown colors, respectively.*

Table S1. *Crystal structure, lattice parameters, and phase composition (wt.%) of fresh HEO samples derived from Rietveld XRD refinement.*

**Sample**	**Phase purity (wt. %,)**	**Lattice (Space group)**	**Lattice parameter**	
			**a (Å)**	**c (Å)**
**HEO(Mg)**	$CuFe_2O_4$: 100	Cubic (Fd-3m (227))	8.374	–
**HEO(Zn)**	$CuFe_2O_4$: 100	Cubic (Fd-3m (227))	8.394	–
**d-HEO(Fe)**	$CuFe_2O_4$: 90.9<br>Fe_2O_3: 9.1	$CuFe_2O_4$: Cubic (Fd-3m (227))<br>Fe_2O_3: Hexagonal (R-3cH (167))	$CuFe_2O_4$:8.367<br>Fe_2O_3 : 5.034	$CuFe_2O_4$: –<br>Fe_2O_3 :13.746

Table S2. *Phase fractions (wt.%) and crystal structures of the reduced HEO samples determined by XRD lattice refinement.*

**Sample**	**Composition in wt. %**			
	**Fe**<br>(Cubic, space group: Im-3m)	**FeNiCu**<br>(Cubic, space group: Fm-3m (225))	**FeO**<br>(Cubic, space group: Fm-3m (225))	**MnO**<br>(Cubic, space group: Fm-3m (225))
**HEO(Mg)**	82.4	5.1	12.5	–
**HEO(Zn)**	88.0	5.1	–	6.9
**d-HEO(Fe)**	88.9	4.3	–	6.8

Table S3. *Raman peak positions and vibrational assignments*

	A_{1g}	$F_{2g(3)}$	$F_{2g(2)}$	E_g	$F_{2g(1)}$
d-HEO(Fe)	677	597	476	330	185
HEO(Mg)	677	601	470	308	185
HEO(Zn)	669	613	475	313	181

Table S4. *ICP measured elemental composition.*

**Element**	**Content (molar %)**			
	**Stoichiometric composition**	**HEO(Mg)**	**HEO(Zn)**	**d-HEO(Fe)**
**Fe**	66.67 (76.67)*	67.99	68.18	76.99
**Ni**	7.00	6.82	6.91	6.90
**Cu**	7.00	6.81	6.82	6.86
**Co**	4.67	4.44	4.50	4.63

**Mn**	4.67	4.50	4.59	4.62
**Mg**	10.00	9.43	–	–
**Zn**	10.00	–	9.00	–

**: stoichiometric concentration of Fe in d-HEO(Fe) sample.*

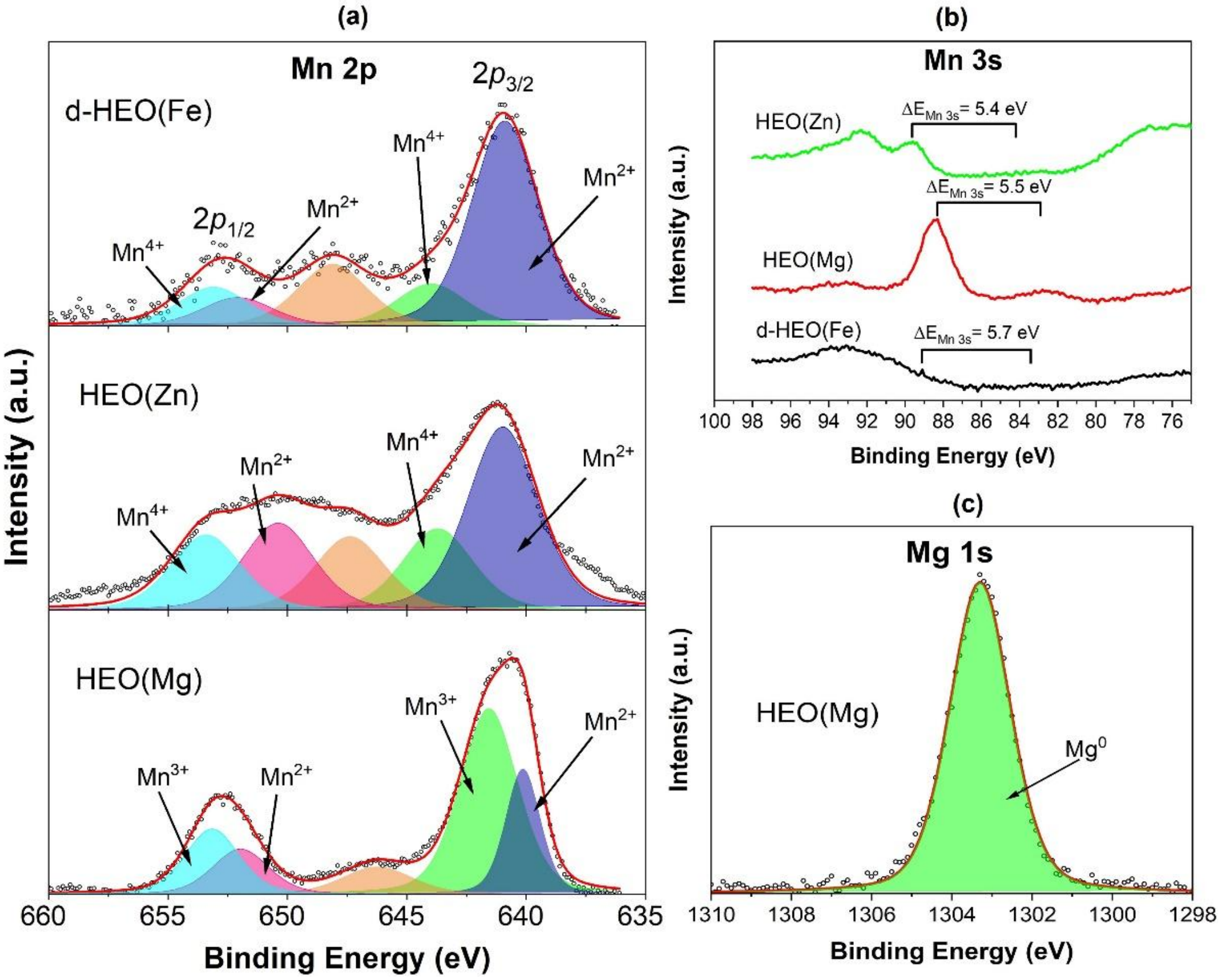

Figure S2. *XPS spectra of the reduced HEO samples; (a) Mn 2p, (b) Mn 3s. High resolution spectra of the (c) Mg 1s for the reduced HEO(Mg) sample.*

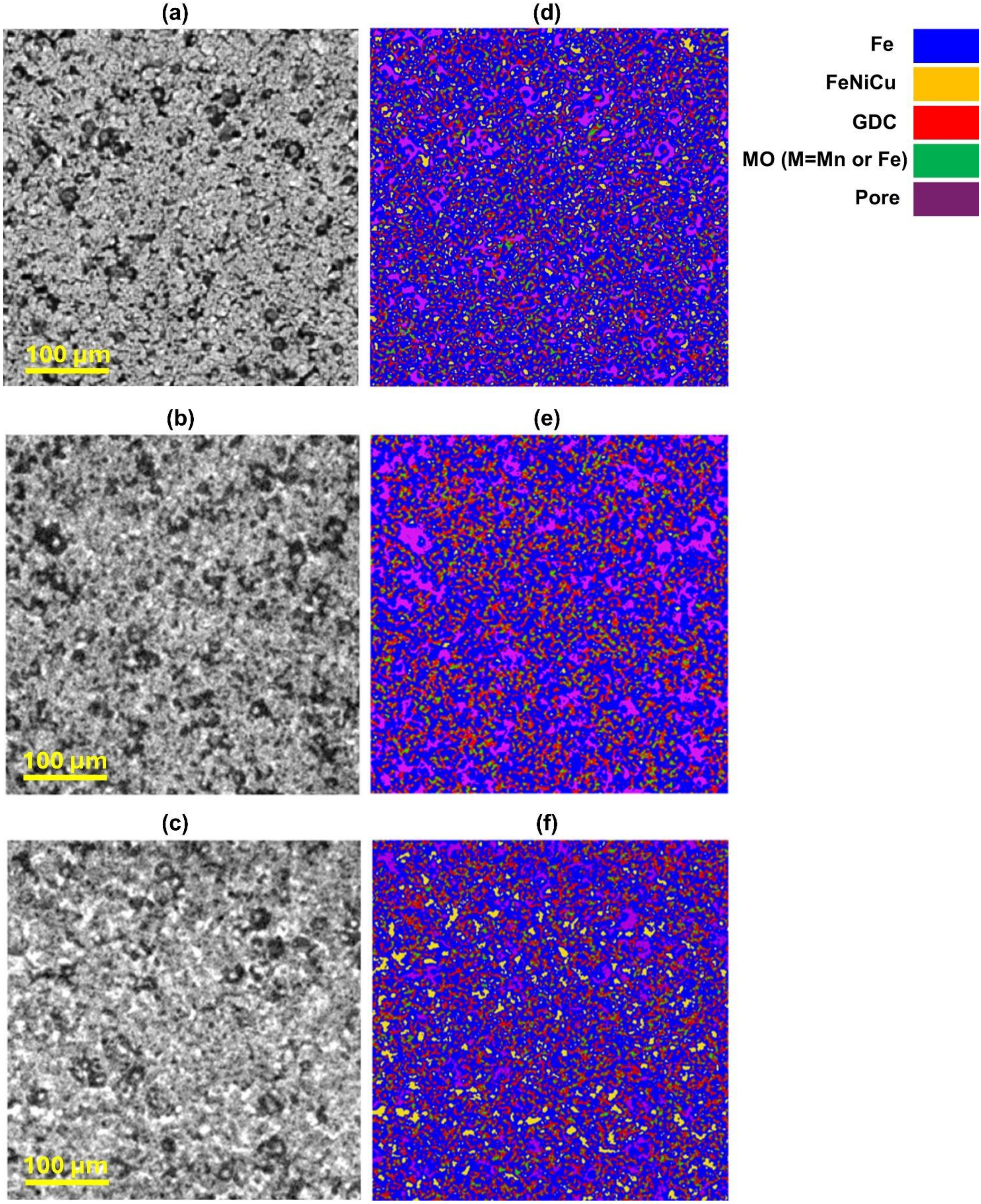

Figure S3. *The raw and segmented 2D slices of the reduced anodes having (a,d) d-HEO(Fe) (b,e) HEO(Zn) and (c,f) HEO(Mg).*

Table S5. *Computed Bader charges of the atoms of the gas-phase and adsorbed ammonia molecule at different sites.*

**Adsorption position**	**Atom**	**NH_3 Adsorbate** *(e)*	**Isolated NH_3 state** *(e)*	**Δq** *(e)*
**Fe atop**	**N**	6.22	6.14	0.08
	**H**	0.57	0.60	-0.03
	**H**	0.57	0.64	-0.07
	**H**	0.58	0.62	-0.03
**Ni atop**	**N**	6.54	6.50	0.04
	**H**	0.47	0.51	-0.04
	**H**	0.46	0.49	-0.04
	**H**	0.46	0.50	-0.04
